# Flexible suspended gate organic thin-film transistors for ultra-sensitive pressure detection

**DOI:** 10.1038/ncomms7269

**Published:** 2015-03-03

**Authors:** Yaping Zang, Fengjiao Zhang, Dazhen Huang, Xike Gao, Chong-an Di, Daoben Zhu

**Affiliations:** 1 Beijing National Laboratory for Molecular Sciences, Key Laboratory of Organic Solids, Institute of Chemistry, CAS, Beijing 100190, China; 2 University of Chinese Academy of Sciences, Beijing 100049, China; 3 Key Laboratory of Synthetic and Self-Assembly Chemistry for Organic Functional Molecules, Shanghai Institute of Organic Chemistry, CAS, Shanghai 200032, China

## Abstract

The utilization of organic devices as pressure-sensing elements in artificial intelligence and healthcare applications represents a fascinating opportunity for the next-generation electronic products. To satisfy the critical requirements of these promising applications, the low-cost construction of large-area ultra-sensitive organic pressure devices with outstanding flexibility is highly desired. Here we present flexible suspended gate organic thin-film transistors (SGOTFTs) as a model platform that enables ultra-sensitive pressure detection. More importantly, the unique device geometry of SGOTFTs allows the fine-tuning of their sensitivity by the suspended gate. An unprecedented sensitivity of 192 kPa^−1^, a low limit-of-detection pressure of <0.5 Pa and a short response time of 10 ms were successfully realized, allowing the real-time detection of acoustic waves. These excellent sensing properties of SGOTFTs, together with their advantages of facile large-area fabrication and versatility in detecting various pressure signals, make SGOTFTs a powerful strategy for spatial pressure mapping in practical applications.

## Abstract (web summary)

The development of wearable healthcare and intelligence systems require low-cost, large-area flexible electronic sensors. Here, Zang *et al*. report pressure sensors based on organic thin-film transistors in combination with a suspended-gate device geometry, which show high sensitivity up to 192 kPa^−1^.

## Graphical abstract

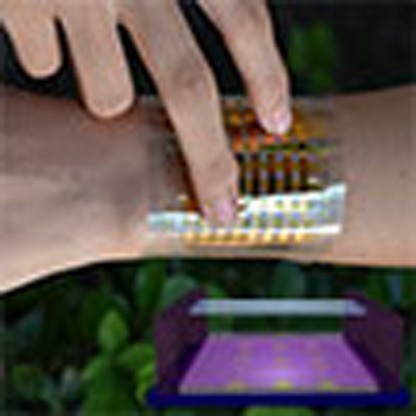

Motivated by the urgent demand for advanced artificial intelligence and wearable healthcare devices, the development of low-cost, flexible electronic sensors is currently attracting profound interest^1^,^2^,^3^,^4^,^5^,^6^,^7^,^8^,^9^,^10^,^11^,^12^,^13^,^14^,^15^,^16^,^17^,^18^,^19^,^20^,^21^,^22^,^23^,^24^,^25^,^26^,^27^,^28^. To meet the critical requirements of these applications, promising routes towards smart devices such as integrated monitoring systems laminated on the human epidermis have been successfully demonstrated in recent years^29^,^30^,^31^,^32^,^33^,^34^. In spite of these achievements, the development of pressure-sensing pixels is still of fundamental importance to the realization of these fascinating applications. As an essential element of various intelligent devices, organic thin-film transistors (OTFTs) not only are distinguished by their excellent flexibility and potential for low-cost, large-area fabrication via solution-processing techniques, but also possess the integrated functionality of signal transduction and amplification^1^,^3^,^6^,^10^,^11^,^12^,^18^,^27^. The combination of these properties means that, in principle, OTFTs are an ideal choice for ultra-sensitive pressure detection applications, including flexible e-skin and healthcare devices^3^,^18^,^35^.

The first attempt to develop an OTFT-based pressure sensor relied on the engineered exploration of organic transistors as electronic readout elements for conductive rubber pressure-sensing components^1^,^6^,^10^,^11^,^12^. The sensing performance, however, was limited by the integrated rubber-based pressure sensors. Taking advantage of the effective strategy of incorporating an organic transistor with a microstructured polydimethylsiloxane (PDMS) dielectric layer^3^,^18^, more recently developed OTFT-based pressure sensors are mechanically flexible and robust, enabling sensitive detection of subtle pressure changes with a high sensitivity of up to 8.4 kPa^−1^ (ref. 18). Despite these pioneering works, organic transistor-based pressure sensors with ultra-high sensitivity (>100 kPa^−1^) have not yet been demonstrated, which is due, in part, to the elastic limitation of the rubber-based dielectric layer.

Given the fundamental mechanism of the reproducible changes that occur in the dielectric layer under pressure biasing in a transistor-based pressure sensor, the application of an air dielectric layer and suspended gate electrodes may intrinsically overcome the elastic limitation of the rubber dielectric layer. The so-called suspended gate metal-oxide-semiconductor field-effect transistors (SGMOSFETs)-based pressure sensors have been demonstrated using conventional inorganic materials^36^,^37^,^38^. However, inorganic SGMOSFETs are not considered to be promising candidates for widely pressure-sensing applications due to the complex high-cost manufacturing processes and unsatisfactory pressure-sensing performance^3^,^38^. Although organic SGMOSFETs can be principally constructed to enable their low-cost and flexible applications, they have not been reported because most organic materials cannot survive the conventional SGMOSFET fabrication procedures. These key issues make the construction of low-cost, high-performance and flexible SGMOSFET-based pressure sensors a great challenge.

Here we demonstrate flexible suspended gate OTFTs (SGOTFTs), which can be constructed using a simple and high-throughput method and can serve as a versatile platform for ultra-sensitive pressure detectors. By fine-tuning the properties of the suspended gate, the SGOTFT can be optimized to achieve an unprecedented sensitivity of 192 kPa^−1^ and can operate with an ultra-low power consumption of <100 nW. The ultra-high sensitivity of SGOTFTs to low pressures even allows for the real-time detection of acoustic waves and wrist pulses, thus enabling their promising application in artificial intelligence and healthcare systems.

## Results

### Basic mechanism and device fabrication

**Figure 1:**
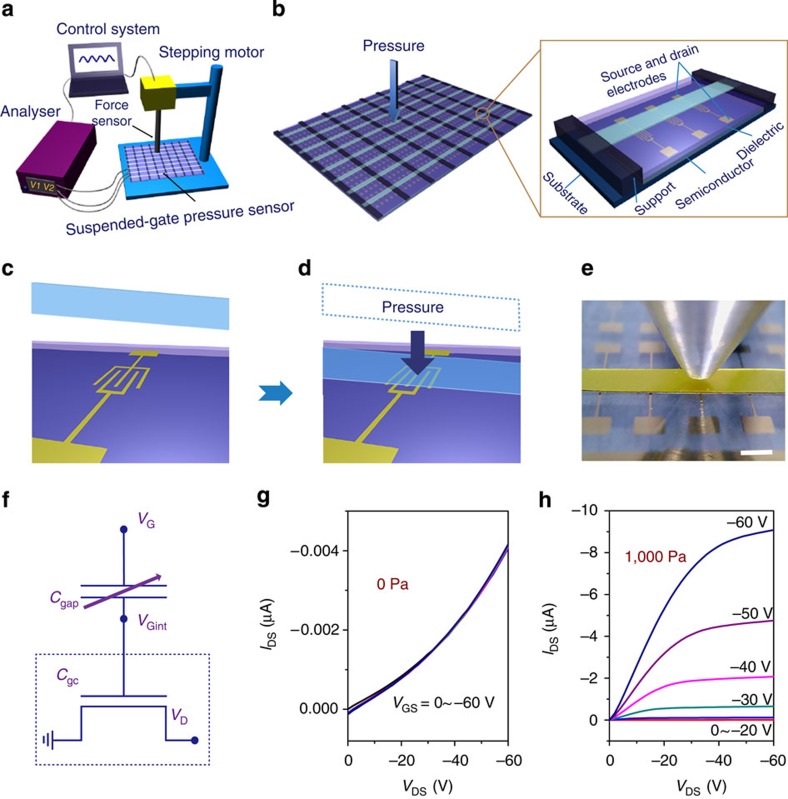
Suspended gate organic thin-film transistor pressure sensors. Schematic illustration of (**a**) the experimental set-up, (**b**) a SGOTFT-based array with magnified device geometry and (**c**) (**d**) pressure-sensing process. (**e**) Photograph of a SGOTFT; scale bar, 1 mm. (**f**) Electrical equivalent circuit of the SGOTFT. Output curves of the SGOTFT taken at a pressure of (**g**) 0 and (**h**) 1,000 Pa.

In an SGMOSFET, the gate deforms when subjected to a force, which results in a change in the capacitance of the dielectric layer as a function of the applied pressure, consequently leading to a pressure-dependent source-drain current at constant gate voltage (Fig. 1a–f)^39^. The sensing performance, therefore, is governed by the properties of the suspended gate and the air gap space. An important strategy of our proposed device is the lamination of a flexible suspended gate onto the device to create a large air gap space of several tens of micrometres, compared with the air gap space of hundreds of nanometres between the rigid gate electrodes and the dielectric layer in a typical inorganic SGMOSFET. This large space enables the facile construction of SGOTFTs.

We first separately deposited gold source/drain electrodes, the semiconductor layer and the protective dielectric layer on a glass or polyimide (PI) substrate. By incorporating a gate-absent OTFT with a laminated support layer (Kapton tape, 40 μm) and a flexible composite gate electrode of polyimide/aluminium foil (PI/Al; Fig. 1b), we constructed an SGOTFT based on PDPP3T (Supplementary Fig. 1) with a poly(perfluorobutenylvinylether) (CYTOP) protective dielectric layer (400 nm) under ambient conditions, as shown in Fig. 1e (see the Methods section for experimental details). Notably, the device fabrication process is scalable and many commercial materials have been utilized as the semiconductors, support layers and gate electrodes without any pretreatment.

### Device-sensing properties

As shown in Fig. 1g, no obvious modulation was observed when no pressure was loaded because of the ultra-low capacitance (0.01 nF cm^−2^; Supplementary Fig. 2, Supplementary Movie 1) of the uncompressed air gap. However, the device displayed a textbook output curve with well-defined linear and saturation regimes under a constant pressure of 1 kPa (Fig. 1h). The electrical response was confirmed by the pressure-loaded transfer curves of the SGOTFT, which indicated a high mobility of up to 0.34 cm^2^ V^−1^ s^−1^ and an on/off ratio of 10^4^ (Supplementary Fig. 3). It should be noted that the fabricated devices exhibited very low hysteresis, even lower than that of typical devices with a SiO_2_ dielectric layer (Supplementary Fig. 4).

**Figure 2:**
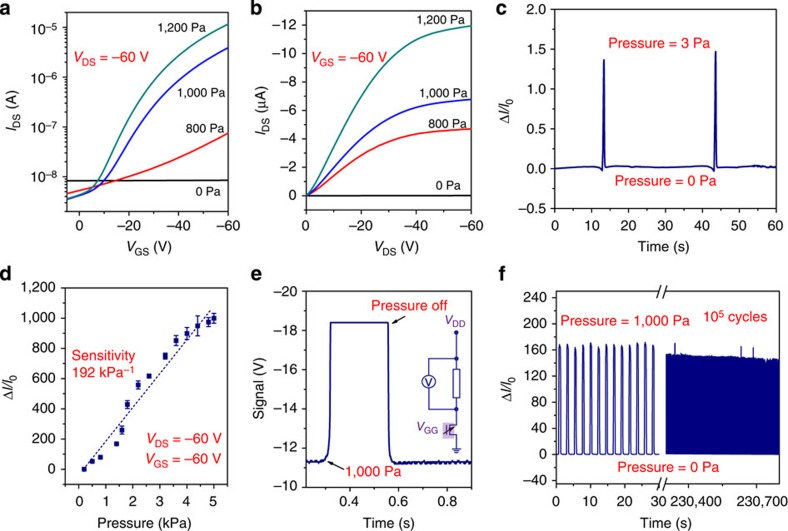
Fundamental electric response and performance data for the SGOTFTs. (**a**) Transfer and (**b**) output curves of a SGOTFT sensor taken at different applied pressures. (**c**) Current response of a SGOTFT to the pressure of 3 Pa. (**d**) Pressure response of the source-drain current at constant voltage *V*_DS_=−60 V and *V*_GS_=−60 V. The error bars represent 1 s.d. (**e**) Oscilloscope recorded time-resolved response of a SGOTFT to a pressure of 1,000 Pa. The inset shows the electrical equivalent circuit of the measurement system. The response and relaxation time are lower than 10 ms. (**f**) The durability test of a SGOTFT under a pressure of 1,000 Pa.

To assess the sensing response of the fabricated devices, we measured the pressure-dependent performance of the SGOTFT. Figure 2a,b presents the transfer and output curves under various applied pressures ranging from 0 to 1.2 kPa. The transistor exhibited a low source-drain current below 10^−8^ A with no pressure load. Increasing the pressure load to 1.2 kPa dramatically increased the on-state current by more than three orders of magnitude, whereas the off-state current slightly decreased (Fig. 2a). Consequently, a high on/off ratio approaching 10^4^ was achieved. From the output curve (Fig. 2b), it can be observed that increasing the pressure resulted in a steadily increasing linear and saturation current. Moreover, the SGOTFT demonstrated an obvious response to a very low pressure of 3 Pa (Fig. 2c), which is the pressure limit of our set-up. In particular, the devices can respond to the loading/unloading of two tiny pieces of paper (0.3 and 0.8 mg weight; Supplementary Fig. 5), which corresponds to pressures of 0.3 and 0.8 Pa, respectively. This sensitive pressure detection, along with the reproducible signal responses to weak air flow fanned by a piece of paper (Supplementary Movie 2), implies an ultra-low limit-of-detection pressure of <0.3 Pa for our fabricated devices.

The sensitivity of our pressure sensor is defined as *S*=(Δ*I*/*I*_0_)/Δ*P*, where Δ*I* is the relative change in current, *I*_0_ is the initial current of the sensor without pressure loading and Δ*P* is the change in applied pressure. By taking advantage of the significant response of the device as a function of the applied pressure, we obtained an ultra-high sensitivity of 192 kPa^−1^ at a constant source-drain voltage (*V*_DS_) and gate voltage (*V*_GS_) of −60 V (Fig. 2d). More importantly, this ultra-high sensitivity was obtained over a wide pressure range from 100 Pa to 5 kPa. To our knowledge, this sensitivity is the highest ever reported among flexible pressure sensors^3^,^4^,^8^,^15^,^18^,^25^,^35^. It should be mentioned that the maximum sensitivity was obtained at a moderate voltage of 60 V, which is obviously lower than that of an organic transistor-based pressure sensor^3^,^18^. We also constructed devices using an alternative semiconducting material, NDI3HU-DTYM2, and a series of protective dielectric layers, including polymethylmethacrylate (PMMA) and polystyrene (PS; Supplementary Figs 1 and 6). All devices displayed excellent pressure-sensing properties, with sensitivities ranging from 26 to 169.2 kPa^−1^ (for *V*_DS_ and *V*_GS_ ranging from 60 to 80 V) and an average value of 92.2 kPa^−1^ (Supplementary Figs 7–9). Therefore, we conclude that the SGOTFT geometry is a generally applicable structure for ultra-sensitive pressure sensors.

Response time is another important parameter for pressure sensors. By virtue of the flexible suspended gate, the SGOTFT can display a rapid response to an applied pressure. By using an oscilloscope to monitor the source-drain current via the change in the voltage drop over a 10-MΩ resistor at a constant *V*_DS_ and *V*_GS_ of −60 V, we observed that the pressure sensor exhibited a virtually instantaneous response to an applied pressure of 1 kPa (Fig. 2e). The measured response and relaxation times were less than 10 ms, which was the threshold value of the stepper motor used in our measurement system. As far as sensitivity and response time are concerned, the pressure-sensing performances of our fabricated SGOTFT were demonstrated to be superior even to those of human skin^20^.

In SGOTFT devices, the *I*_DS_ in the saturated regime can be described as follows (a detailed discussion is provided in Supplementary Note 1):


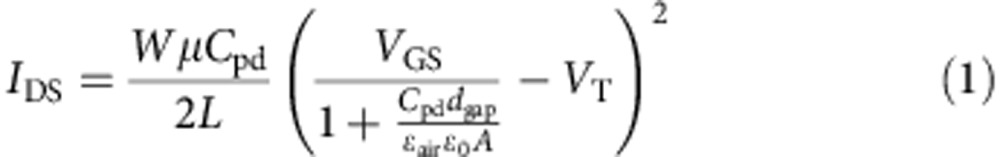


where *I*_DS_ represents the drain current, *L* and *W* represent the channel length and width of the OTFT device, respectively, *μ* is the carrier mobility, *V*_T_ is the threshold voltage, *C*_pd_ is the capacitance of the protective dielectric layer, *ε*_0_ is the absolute dielectric constant, *ε*_air_ is the relative dielectric constant of air, *A* is the area of overlap between the gate and the device and *d*_gap_ is the thickness of the air gap. It is evident that *I*_DS_ is highly dependent on *d*_gap_ for a given device. As the suspended gate can be bent into a particular curvature under pressure, *d*_gap_ can change as a function of the applied pressure (see the Supplementary Note 1), which can lead to dramatic changes in the capacitance of the dielectric layer. For example, the relative change in capacitance (Δ*C*/*C*_0_) can reach 12 when the device is subjected to a pressure of 5 kPa (Supplementary Fig. 2). This value is markedly higher than that which can be achieved in a microstructured PDMS (Δ*C*/*C*_0_<2)^3^,^18^. This dramatic enhancement of the capacitance and the transistor dominated signal amplification^3^,^18^, therefore, are responsible for the ultra-high sensitivity of our fabricated SGOTFT. As the protective dielectric layer does not change on external pressure and an SGOTFT without a protective dielectric layer exhibits reproducible responses to pressure loads (Supplementary Fig. 10), we can draw the meaningful conclusion that the pressure-induced changes in capacitance are governed by the mechanical properties of the suspended gate (Supplementary Note 1).

### Tunable sensing performance

**Figure 3:**
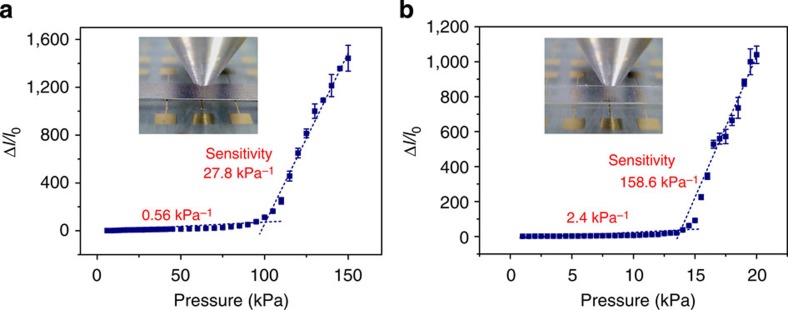
Tunable pressure-sensing performance. Pressure sensitivity of the (**a**) stainless steel gate and (**b**) PET/ITO gate-based SGOTFTs, respectively, at constant *V*_DS_=−60 V and *V*_GS_=−60 V. The error bars represent 1 s.d.

In general, different sensing applications require varied sensitivities in different pressure regimes. Therefore, the tuning of sensing performance is an important but challenging task^18^. As mentioned above, the mechanical properties of the suspended gate in an SGOTFT can affect the device’s capacitance response to a pressure load. Therefore, the modulation of the suspended gate can serve as an excellent strategy for the modulation of the sensing performance of the SGOTFT. The vertical displacement of the suspended gate under an external pressure is predominantly influenced by the rigidity (*k*) of the gate (a detailed discussion is provided in Supplementary Note 1), which is determined by its geometry and dimensions as follows^40^:


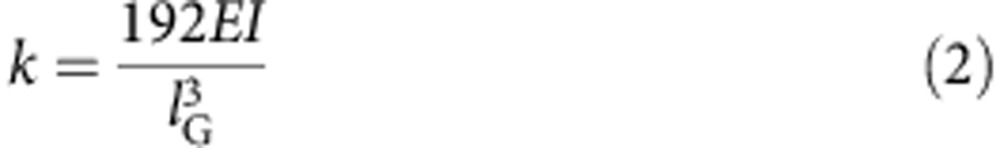



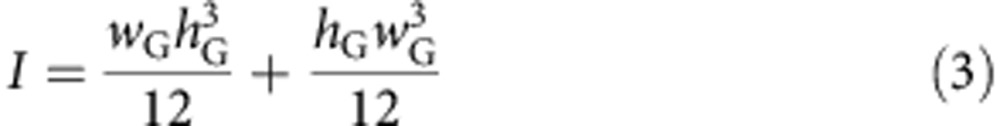


where *I* is the bending moment of inertia of the rectangular gate, *E* is the Young’ s modulus and *h*_G_, *l*_G_ and *w*_G_ are the gate thickness, length and width, respectively. According to these equations, adjusting the modulus and/or dimensions of the suspended gate offers an effective method of tuning the sensitivity and response range of the SGOTFTs. We first investigated the suspended gate-dependent sensing performance of SGOTFTs by employing various conducting materials as the suspended gates. As expected, devices with different suspended gates exhibited different current responses as a function of pressure. When a 100-μm stainless steel sheet was used as the suspended gate, we obtained a moderate sensitivity of 27.8 kPa^−1^ for pressure load greater than 100 kPa (Fig. 3a). Because a much higher sensitivity of >150 kPa^−1^ can routinely be obtained when a PET/indium-tin-oxide (ITO) or PI/Al film is used as the suspended gate (Figs 2d and 3b), we conclude that a flexible suspended gate with a low modulus^41^ is preferred to achieve an ultra-high sensitivity in the low-pressure regime. The influence of the gate thickness on the sensitivity was then examined by utilizing stainless gates with different thicknesses. Thinner stainless steel (30 μm) resulted in a significantly increased sensitivity (70.2 kPa^−1^; Supplementary Fig. 11), in good agreement with equations (2) and (3). This unique feature of SGOTFTs could allow for the modulation of their sensitivity in accordance with the needs of the desired pressure regime.

**Figure 4:**
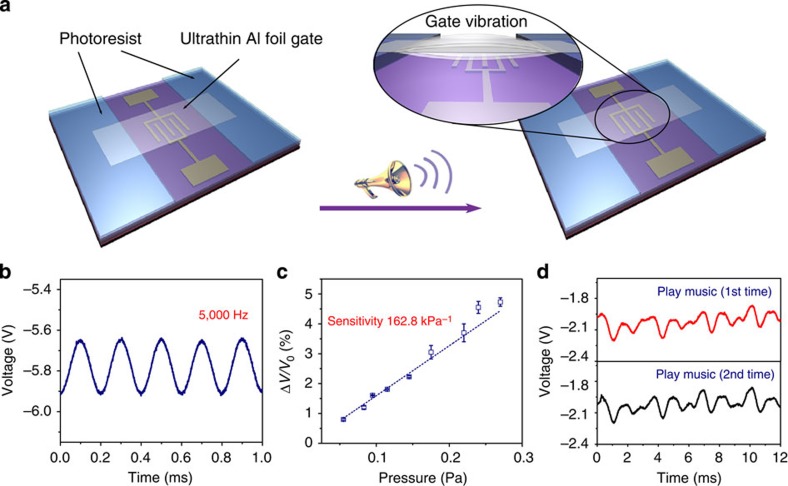
The SGOTFT in acoustic wave detection. (**a**) Schematic illustration of device geometry for ultra-sensitive detection of acoustic wave. (**b**) Response of a sensor, integrated with 4 μm photoresist and 10 μm Al foil suspended gate, to the acoustic wave with a fixed frequency of 5 kHz. (**c**) Sound pressure sensitivity of SGOTFT. The error bars represent 1 s.d. (**d**) Electric signal response of a sensor to the same music for two times.

When a laminated ultra-thin Al foil is used as the suspended gate, ultra-low-pressure changes can cause the vibration of the suspended gate. These vibration-induced electrical signals are dramatically amplified when the air gap is a few micrometres in size. For example, when the thickness of the Al foil and the air gap space were chosen to be 10 and 4 μm (Supplementary Fig. 12), respectively, the electrical performance of the SGOTFT was influenced by acoustic wave, thus enabling the sensitive detection of acoustic waves (Fig. 4a). Figure 4b presents the signal response of the SGOTFT to an acoustic wave with a frequency of 5 kHz (80 db). In this manner, we determined that the devices exhibited a short response time of 0.2 ms, which may be attributed to the avoidance of the viscoelastic behaviour that is typical of a rubber dielectric layer. Moreover, the signal response increased linearly from 0.05 to 0.3 Pa with a high sensitivity of 162.8 kPa^−1^ (Fig. 4c, Supplementary Note 2). The rapid response time of <1 ms, ultra-high sensitivity of >150 kPa^−1^ and low limit-of-detection pressure of <0.1 Pa allowed for repeatable, real-time detection of different types of music sounds (Fig. 4d, Supplementary Movie 3, Supplementary Fig. 13).

### Operating stability

To assess the possible influence of the air gap on the device stability, an SGOTFT durability test was performed. The device displayed a steady current response (Δ*I*/*I*_0_) of 160 to a pressure of 1 kPa. Interestingly, the sensor performance exhibited a slight change after 10^5^ cycles of loading-unloading tests for both *p*- and *n*-channel devices (Fig. 2f and Supplementary Fig. 14). In addition to operating stability, temperature-induced shifts also pose important challenge in the use of SGMOSFETs for pressure sensing. In a previously reported SGMOSFET based on inorganic materials, a large thermally induced signal shift (4 kPa K^−1^) has been observed during sensing processes of static pressure, raising concerns regarding reliable low-pressure sensing^3^,^38^. Our organic-materials-based SGOTFT, however, exhibited a smaller thermal signal shift of only 0.04 kPa K^−1^ taken at a pressure of 3 kPa (Supplementary Fig. 15). This signal shift was significantly smaller than that reported for the inorganic SGMOSFET and comparable with that of the OFETs based sensor with microstructured PDMS^3^,^38^. This considerably reduced thermal shift can most likely attribute to the moderately temperature-dependent mobility and the open air gap geometry of our SGOTFTs. Although the open air gap dielectric structure can principally make the device dependent on the environmental conditions such as humidity, the problem can resolved by a tight encapsulation of polymeric films such as PI and so on. (Supplementary Fig. 16).

### Battery-driven measurements of wrist pulses

**Figure 5:**
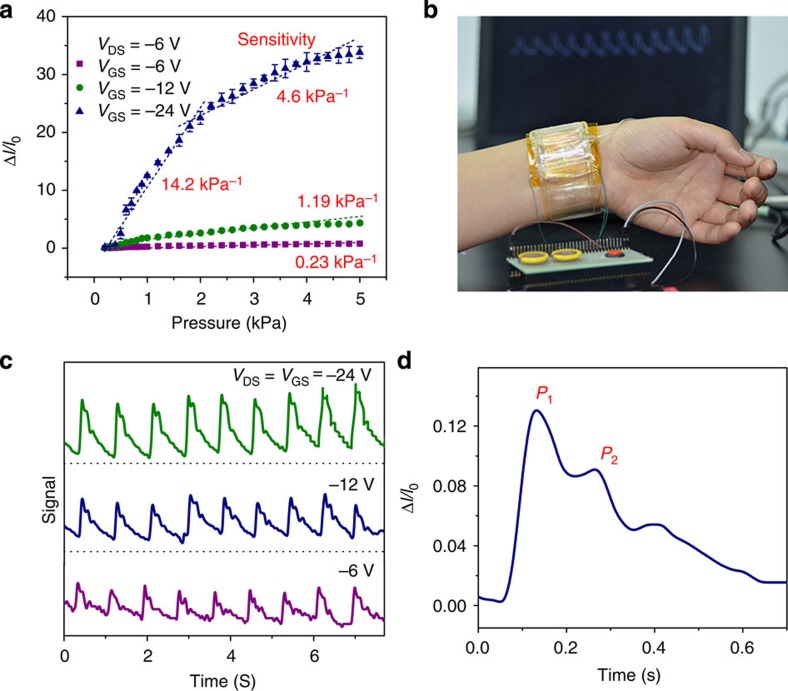
Pulse wave measurement. (**a**) Pressure sensitivity of the SGOTFT device under different *V*_GS_. The error bars represent 1 s.d. (**b**) Photograph of battery-powered SGOTFT attached to the artery of the wrist. (**c**) The signal curves of real-time pulse wave under different voltages. (**d**) A magnified curve of average pulse wave signal from five devices (separate measurement): P1, P2 and diastolic wave are observed clearly.

The ability to operate at low voltage is vital for the practical application of SGOTFT-based sensors to ensure their low power consumption and efficient integration with power storage units in wearable electronics. Despite the large air gap in our SGOTFT, we found that the device could operate well even at low voltages below 20 V when the thickness of the protective layer was decreased to 50 nm. It should be noted that the source-drain voltage exerts a limited effect on the pressure-sensing performance (Supplementary Fig. 17a), whereas the gate voltage influences the signal response to a much greater extent (Supplementary Fig. 17b). We measured the *V*_GS_-dependent sensitivity using a fixed, battery-compatible *V*_DS_ of −6 V (Fig. 5). In contrast to the high sensitivity (4.6–14.2 kPa^−1^) measured at a *V*_GS_ of 24 V, the sensitivity decreased to 0.23 kPa^−1^ at a *V*_GS_ of 6 V (Fig. 5a). This sensitivity at *V*_GS_<30 V, however, is comparable to that of many prominent pressure sensors^3^,^8^,^19^,^23^,^25^,^35^ and is sufficient for the detection of the radial artery pulse, which exerts a typical pressure of ~15 kPa. In particular, the low power consumption of <1 mW for the SGOTFT that operates with a maximum sensitivity >190 kPa^−1^ (*I*_DS_<10 μA @ *V*_DS_=−60 V) is significant. When the device operates at a low voltage of −6 V, an ultra-low power consumption of <100 nW can be achieved for our device (*I*_DS_<15 nA @ *V*_DS_=−6 V). This suggests that SGOTFT-based pressure sensors are potentially applicable for long-term service in wearable applications.

Given the good flexibility of our SGOTFT (Supplementary Figs 18 and 19) and its ability to operate in the low-voltage regime, we were able to successfully construct an SGOTFT driven by two batteries (6 V; Fig. 5b). A typical pulse pressure shape was obtained, with three clearly distinguishable peaks (Fig. 5c,d, Supplementary Movie 4)^18^,^42^. From the P1 and P2 waves, we could easily derive two of the parameters that are most commonly used for health monitoring, namely, the radial augmentation index AI_r_=P2/P1 and Δ*T*_DVP_=*t*_t_−*t*_p_. We calculated values for these parameters of 0.60 and 135 ms, respectively, which are characteristic values for a healthy adult male.

### Pressure sensor array

**Figure 6:**
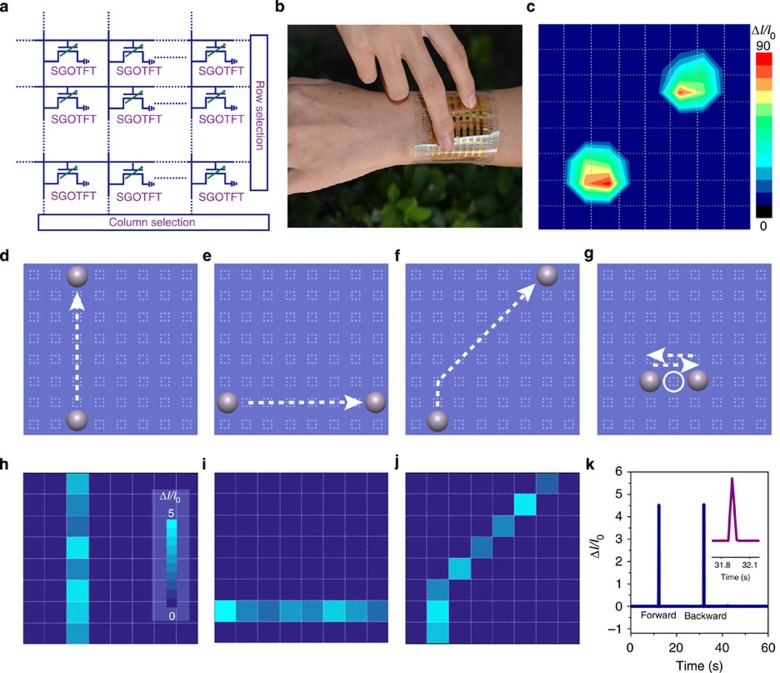
Photographs and performances of SGOTFT pressure-sensing array. (**a**) Circuit schematic of the pressure-sensing array. (**b**) A photograph and (**c**) resulting current mapping of a flexible 8 × 8 pressure-sensing array to the subtle touches, the array with a total area of 6 × 6 cm^2^ and each pixel has an area of ~2.5 mm^2^; scale bar, 5 cm. Schematic illustrations of (**d**–**f**) corresponding current mappings (**h**–**j**) a ball (80 mg) moving along different paths. Illustration image (**g**) and corresponding current response (**k**) to the back and forth movement of the ball on s single device. The inset shows the magnified response signal.

To meet the requirements of various artificial intelligence and healthcare applications, it is desirable to build sensors into a device array to realize a spatially resolved sensing element. As a demonstration, we fabricated a flexible 8 × 8 proof-of-concept sensor array of 6 × 6 cm^2^ (~2.5 mm^2^ area of each pixel, Supplementary Fig. 20) with integrated SGOTFT on a PET substrate (20 μm in thickness; Fig. 6a, the fabrication processes are described in detail in the Methods section). All devices exhibited typical transistor behaviour at *V*_DS_ and *V*_GS_ of 60 V under pressure biasing. By virtue of the good uniformity and excellent flexibility of the devices (Supplementary Fig. 21), the array was able to produce spatially resolved images with subtle imaging features. Figure 6b shows a wearable sensing array attached to the wrist of an adult woman, and the contact points were identified by measuring the pressure on a reconstructed map, as shown in Fig. 6c, where the colour of each pixel bar corresponds to the contact pressure on the sensing array. More interestingly, motion monitoring was achieved using the pressure-sensing array. When a ball (80 mg, with a radius of 2 mm) moves along the sensing array (Fig. 6d–f), the motion path could be clearly recorded (Fig. 6h–j), and the motion speed can be calculated from the signal response. A speed of 7 mm s^−1^ was calculated from the current response of a single device (Fig. 6g,k), consistent with the actual speed of the ball (7.8 mm s^−1^). In the future, it may be possible to construct transparent sensing arrays for promising applications in wearable devices and e-skin, as high-transparency SGOTFT sensing devices on flexible substrates have already been successfully demonstrated using ITO as the source-drain and gate electrodes (Supplementary Fig. 22).

## Discussion

The construction of SGOTFTs is not intended merely for the replacement of inorganic semiconductors in SGMOSFETs with their organic counterparts, but rather opens up new opportunities for low-cost, ultra-sensitive and flexible pressure-sensing applications. By incorporating a flexible suspended gate into an OTFT using a simple lamination method, the facile SGOTFT construction has been achieved. The concept of utilizing an air dielectric layer and suspended gate electrode represents an interesting strategy towards ultra-sensitive flexible pressure sensor. In fact, Bao and colleagues^3^,^18^ proposed the application of air gap dielectric to increase the pressure-sensing performance by using a microstructured PDMS dielectric layer in OTFTs. Despite an impressive sensitivity of up to 8.4 kPa^−1^, realization of ultra-high sensitivity is limited by the elastic properties of the microstructured rubber. The pressure-sensing performance of SGOTFT, however, is determined by the mechanical properties of the suspended gate. As a result, SGOTFTs provide an ideal model platform that enable ultra-sensitive, while tunable, pressure detection.

We have demonstrated that various semiconductors (PDPP3T, NDI3HU-DTYM2) and protective dielectrics (CYTOP, PMMA, PS) can be utilized to fabricate SGOTFTs, thereby demonstrating that the proposed device geometry and fabrication method are generally applicable to different materials. Because the device fabrication processes are compatible with various printing techniques such as roll-to-roll and inkjet printing and so on, all-solution-processed SGOTFTs can be produced in this fashion. In addition to their efficient fabrication procedures, the investigated SGOTFTs displayed unprecedented pressure-sensing performance. An ultra-high sensitivity of 192 kP^−1^ (60 V) in the low-pressure regime of <5 kPa was obtained by using a flexible PI/Al foil as the suspended gate. Notably, even with a large air gap distance, the SGOTFTs can operate at low *V*_DS_ and *V*_GS_ (6–24 V) and exhibit low power consumption ranging from 90 nW to 1 mW while maintaining moderate sensitivity (0.23–14.2 kPa^−1^). The ultra-high sensitivity of SGOTFTs, along with their tunable performance controlled by the mechanical properties of gate electrodes and good operational ability under low voltage, make our SGOTFT among the best flexible pressure sensors ever reported^3^,^4^,^8^,^15^,^18^,^25^,^35^.

The outstanding sensing performance and good flexibility of SGOTFTs allow many novel and fascinating applications of SGMOSFETs. For instance, their remarkable performance has enabled the first realization of an acoustic wave sensor based on organic transistors. The reproducible response of SGOTFTs to an acoustic wave with a high frequency of 5 kHz suggests their potential applications in organic microphone and acoustic communication. Battery-driven, high-fidelity measurement of human radial artery pulse waves has also been realized, in a form that fulfills the requirements for long-term healthcare monitoring. Moreover, touch mapping and motion capture have been demonstrated using an integrated array of flexible SGOTFTs, thereby illustrating the potential application of these devices in wearable electronic elements.

To get the true benefits of wearable electronic elements, the sensing devices should possess excellent flexibility, good scalability and facile integration ability with power supply and/or wireless communication units^29^,^30^,^31^,^32^,^33^,^34^. Further development of SGOTFT will aim at the improvement of scalability and further integration of high-resolution circuits towards their low-cost wearable applications. Construction of all-solution-processed SGOTFTs and corresponded matrix are underway to address these issues. The achievement of these purposes, together with unique features of SGOTFTs may enable the broader application of SGMOSFETs in a variety of emerging areas.

In conclusion, flexible SGOTFTs, which can be constructed using a facile yet efficient approach, provide a powerful strategy for the development of low-cost ultra-sensitive pressure sensors. Notably, the SGOTFT features an ultra-high sensitivity of 192 kPa^−1^, a fast response time of <10 ms and a low power consumption of <100 nW when operated under a battery voltage of 6 V, enabling real-time response to radial artery pulse and acoustic vibrations. This methodology offers a new approach to the use of organic transistors as promising intelligent elements in future wearable electronics, including continuous health-monitoring devices and robotics.

## Methods

### Device fabrication

Glass (Dow Corning), PI (Kapton HN) and polyethylene terephthalate (PET, Mylar) substrates were cleaned with deionized water, ethanol and acetone, respectively, and were then blown dry using a nitrogen gun. Gold source-drain electrodes (30 nm) were deposited on the substrate via vacuum evaporation through a shadow mask at a pressure of 10^−5^ Pa and a rate of 0.4 Å s^−1^. The channel lengths were 50 and 100 μm, and the channel width was fixed to 4,800 μm. Semiconductor solutions of PDPP3T (Solarmer Co., 5 mg ml^−1^, toluene solvent) and NDI3HU-DTYM2 (4 mg ml^−1^, chloroform), which were synthesized following a previously reported method^43^, were spin coated at a speed of 5,000 r.p.m. The films thus obtained were thermally annealed at 150 °C on a hot plate for 5 min. CYTOP (1:3 volume ratio, CT-SOLV 180), PMMA (80 mg ml^−1^, butyl acetate) or PS (40 mg ml^−1^, butyl acetate) was spin coated at a speed of 2,000 r.p.m. to serve as a protective dielectric layer, followed by thermal treatment (100 °C for 20 min on a hot plate) to remove the solvent. Strips of PI tape of 40 μm in thickness were laminated onto the substrates as supports, onto which the PI/Al foil, ITO/PET or stainless gate was transferred and fixed with tapes.

For flexible-array fabrication, the PET substrate (25 μm in thickness) was cleaned as described above, and a shadow mask was then utilized to deposit the source-drain electrodes for a 8 × 8 array with a total area of 6 × 6 cm^2^. The channel width and length of a pixel was 8,500 μm and 80 μm, respectively, and the area of each pixel is ~2.5 mm^2^. PDPP3T (5 mg ml^−1^, toluene solvent) and PMMA (80 mg ml^−1^, butyl acetate) solutions were then spin coated onto the substrates as the semiconductor and protective dielectric layers, respectively. PI tape was then laminated onto the treated substrate to serve as supports, and a PET film (25 μm in thickness) patterned with Au (50 nm in thickness) was finally transferred onto the supports to serve as the suspended gate.

For the fabrication of the SGOTFT-based acoustic sensor, the glass substrate was cleaned and the PDPP3T and CYTOP were deposited as described above. Thereafter, negative photoresist (RPN-1150) was spin coated onto the CYTOP dielectric layer, followed by the application of a typical photolithography procedure to form a patterned support layer based on the photoresist (4 μm in thickness). An ultra-thin aluminium foil (10 μm in thickness) was transferred and fixed onto the support layer to serve as the suspended gate.

### Electrical characterization

Transistor and sensing measurements were conducted using an Agilent B2902 precision source/measure unit under ambient conditions at room temperature. For the dynamic pressure measurement, a force gauge (Mark-10 025/012) and a highly configurable motorized stand (EMS301-CP) were used to apply an external pressure at speeds of 0.5–1100, mm min^−1^. The contact area between the pressing tip and the device is 1.13 mm^2^, which is used to calculate the applied pressure. Capacitance measurements were performed using a Keithley 4200 SCS. A computer-controlled home-made speaker served as an acoustic source, from which both music and acoustic waves of various frequencies could be produced. An oscilloscope (Agilent DSO-X 3052A) was used to record the acoustic sensing signal by measuring the change in voltage drop over a 10-MΩ resistor, which was connected to the SGOTFT acoustic sensor in series. The surface morphologies of the active films were characterized via atomic force microscopy using a Veeco Dimension 3100 (Digital Instruments) in tapping mode. The air gap of the SGOTFT-based acoustic sensor was measured via field emission scanning electron microscopy (Hitachi S-4800, 1 kV, Hitachi, Tokyo, Japan).

## Author contributions

C.-a.D. and D.Z. initiated the study. Y.Z. and C.-a.D. carried out the experiments and analysed the data. F.Z. and D.H. discussed the results. X.G. provided the NDI3HU-DTYM2. Y.Z., C.-a.D. and D.Z. prepared the manuscript. All authors discussed, revised and approved the manuscript.

## Additional information

**How to cite this article:** Zang, Y. *et al*. Flexible suspended gate organic thin-film transistors for ultra-sensitive pressure detection. *Nat. Commun.* 6:6269 doi: 10.1038/ncomms7269 (2015).

## Supplementary Material

Supplementary Figures, Supplementary Notes and Supplementary References Supplementary Figures 1-22, Supplementary Notes 1-2 and Supplementary References

Supplementary Movie 1 Output and transfer curve measurements of a SGOTFT sensor without and with load pressure.

Supplementary Movie 2 Electric response of a SGOTFT to weak air flow fanned by a piece of paper.

Supplementary Movie 3 Electric response of a SGOTFT to an acoustic wave with a frequency of 5 kHz and the sound of piano.

Supplementary Movie 4 Pulse wave measurement using a flexible SGOTFT driven by two batteries (6 V).
