# Supplementary material for: Flexible suspended gate organic thin-film transistors for ultra-sensitive pressure detection
Source: Nat Commun. 2015 Mar 3;6:6269. doi: 10.1038/ncomms7269 (PMC4366495; doi:10.1038/ncomms7269)
Supplement: Supplementary Figures, Supplementary Notes and Supplementary References — Supplementary Figures 1-22, Supplementary Notes 1-2 and Supplementary References [file ncomms7269-s1.pdf]

Supplementary Figures

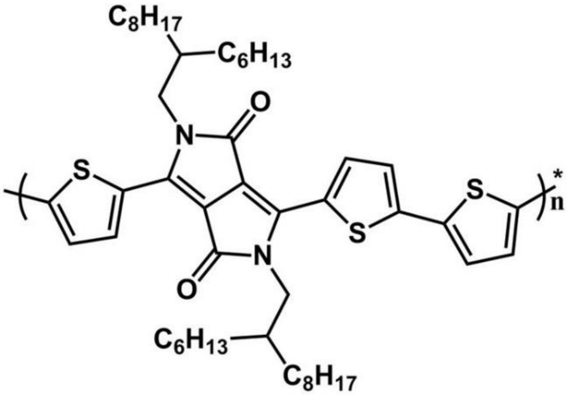

PDPP3T

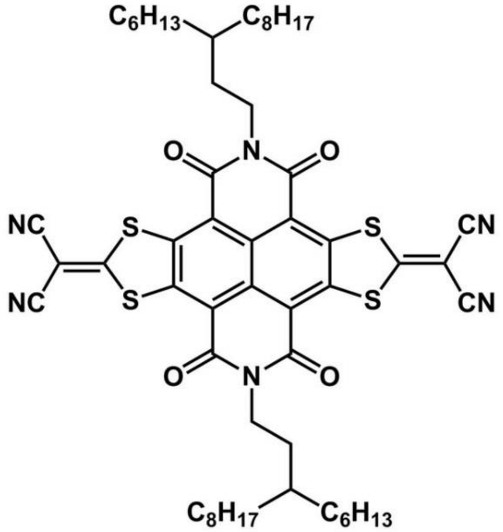

NDI3HU-DTYM2

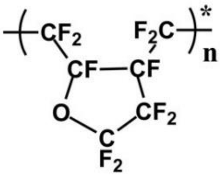

CYTOP

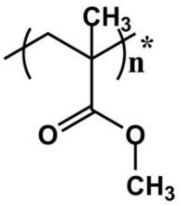

PMMA

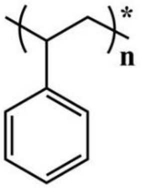

PS

Supplementary Figure 1 | Molecular structures of functional materials involved in our SGOTFT devices.

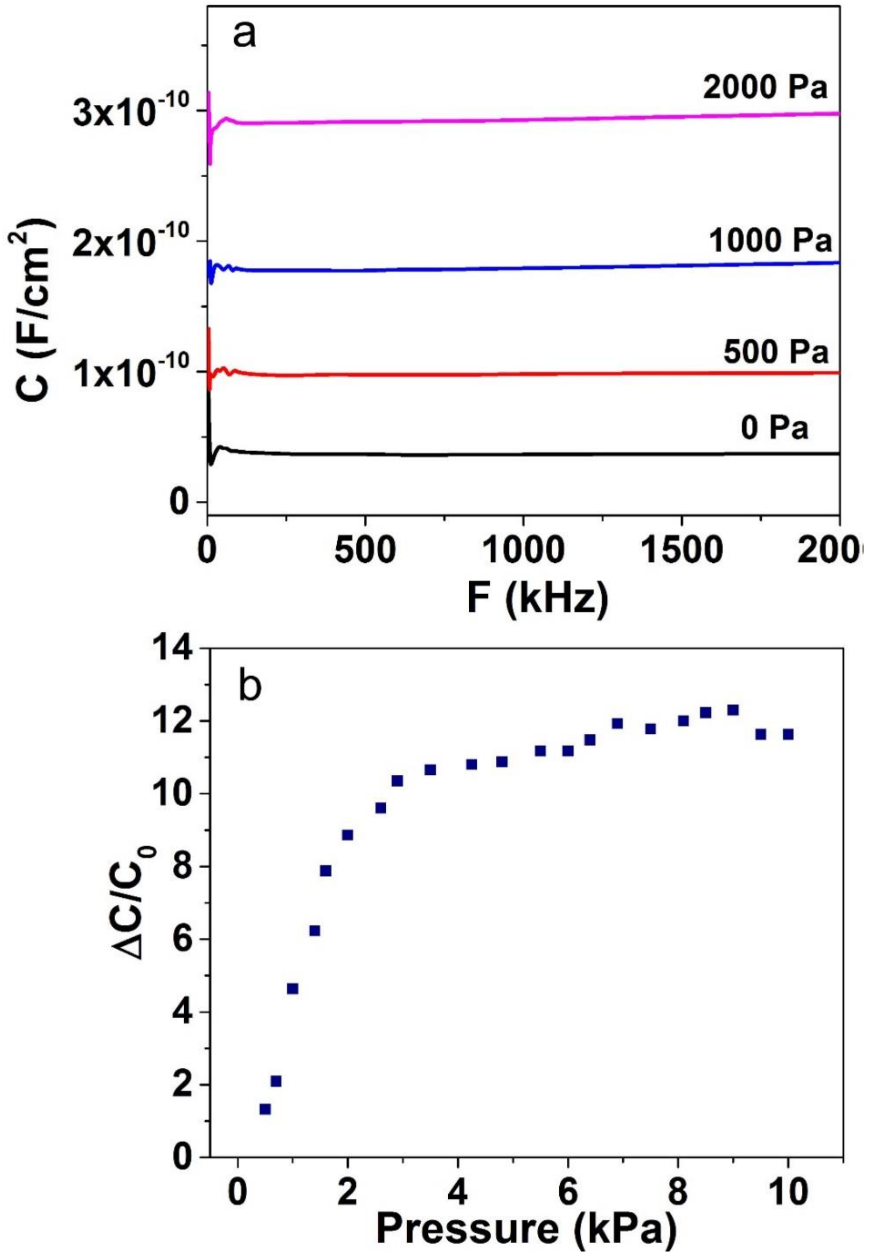

Supplementary Figure 2 | Capacitance measurements of a SGOTFT device. (a) Capacitance curves of a SGOTFT sensor taken at different pressures. (b) Pressure response of the dielectric capacitance measured at 10 kHz.

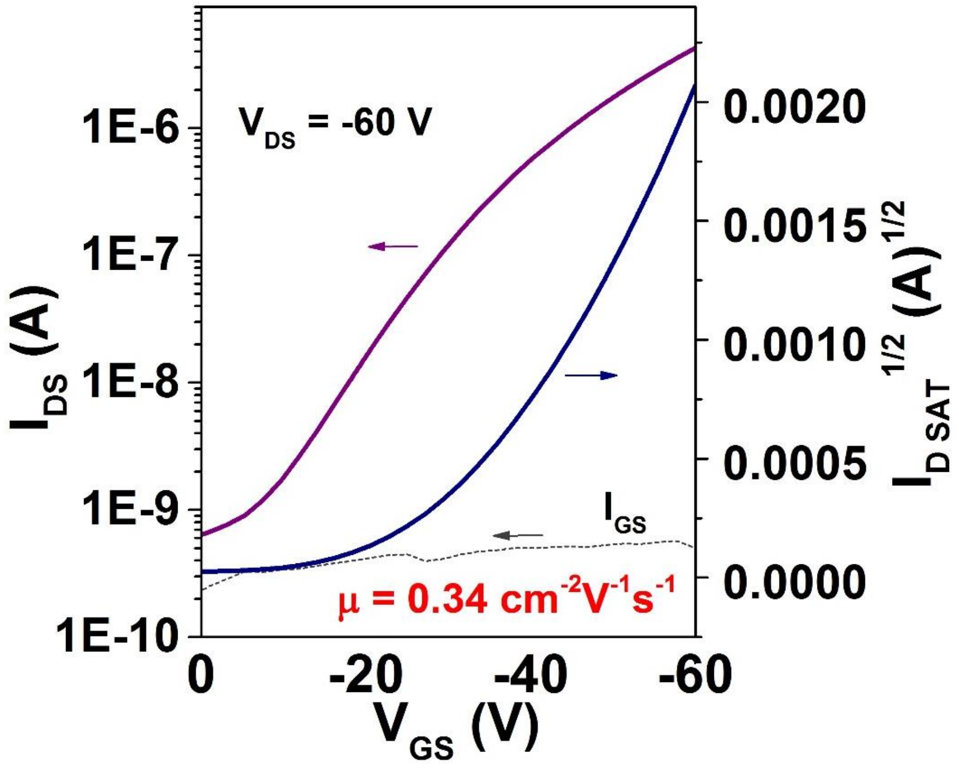

Supplementary Figure 3 | Device performances of PDPP3T based SGOTFTs. Transfer curves of PDPP3T based SGOTFTs taken at a pressure of 1000 Pa.

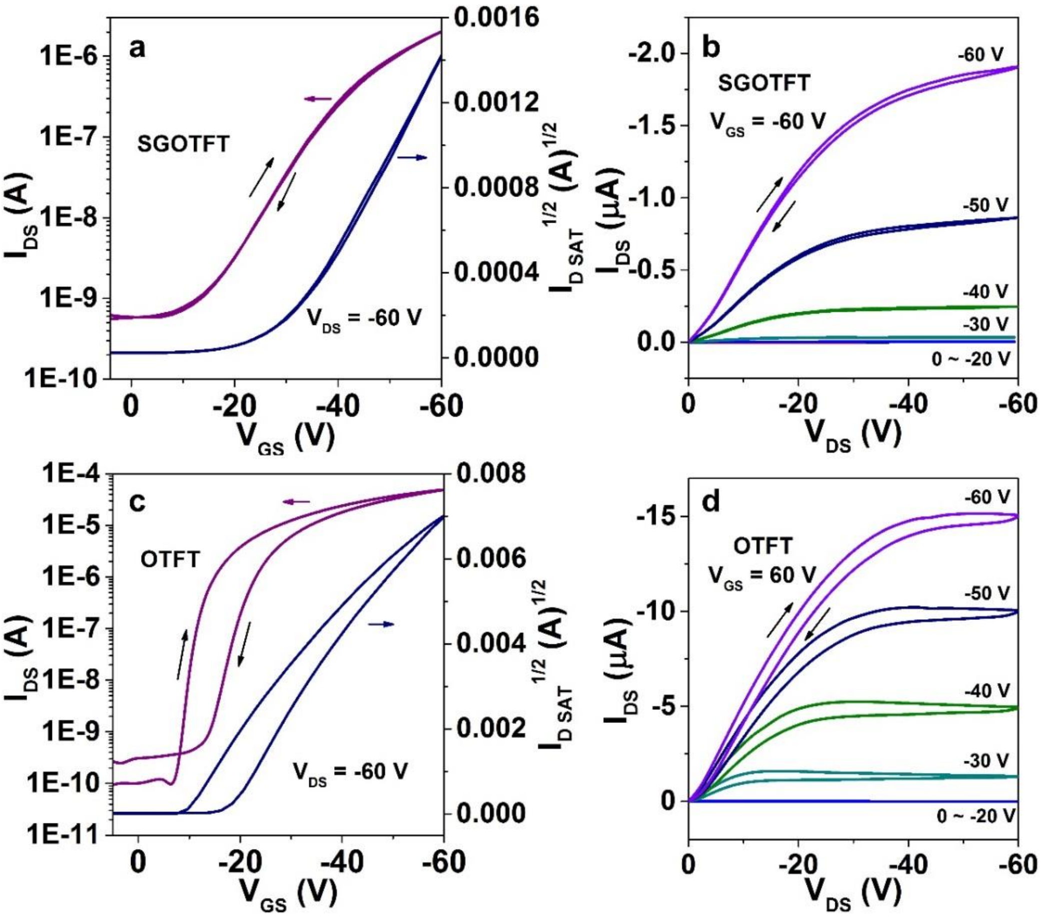

Supplementary Figure 4 | Device performances of PDPP3T based SGOTFT and classic OTFT devices. (a) Transfer and (b) output curves of a SGOTFT taken at a pressure of 2000 Pa. (c) Transfer and (d) output curves of the bottom gate bottom contact OTFT.

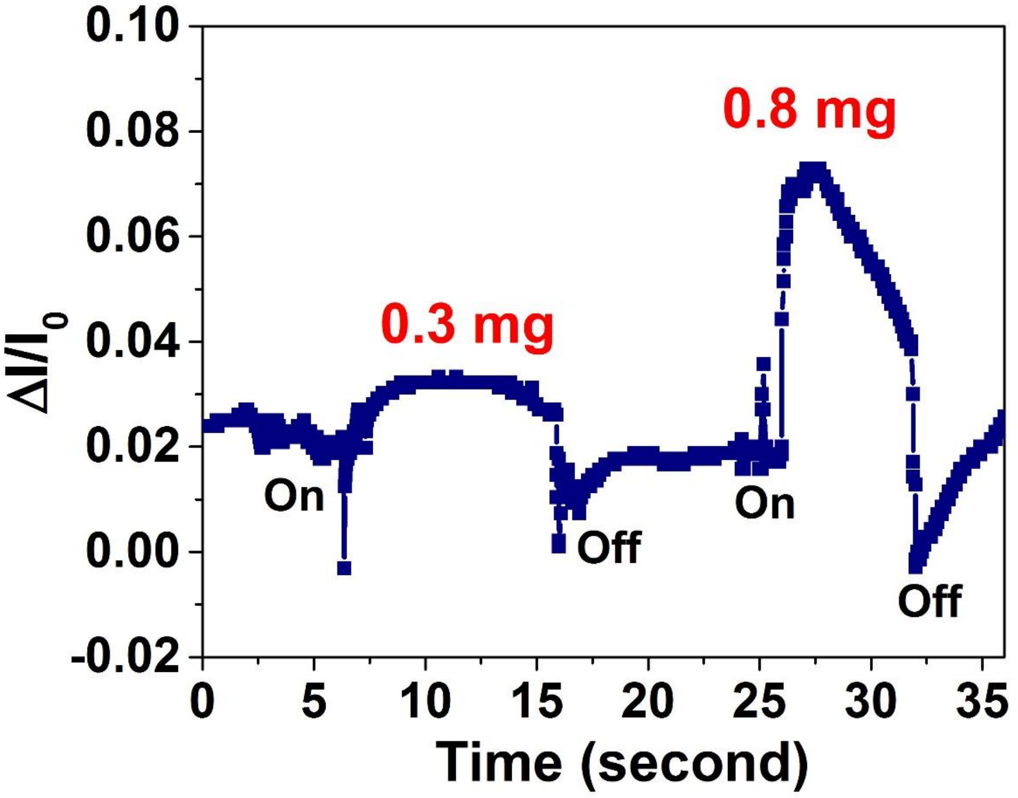

Supplementary Figure 5 | Electric responses of a SGOTFT to the loaded and unloaded piece of paper of a 0.3 mg and 0.5 mg weight, respectively.

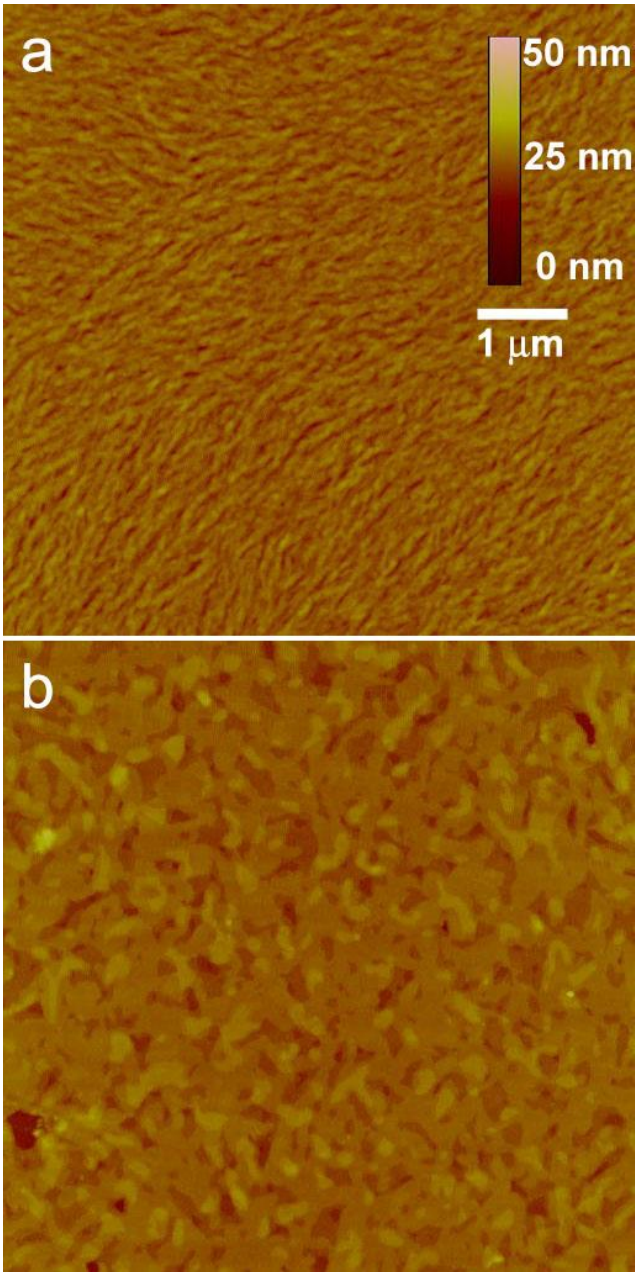

Supplementary Figure 6 | Morphology characterizations of various semiconductors based SGOTFTs. AFM images of (a) PDPP3T and (b) NDI3HU-DTYM2 films on the glass substrate.

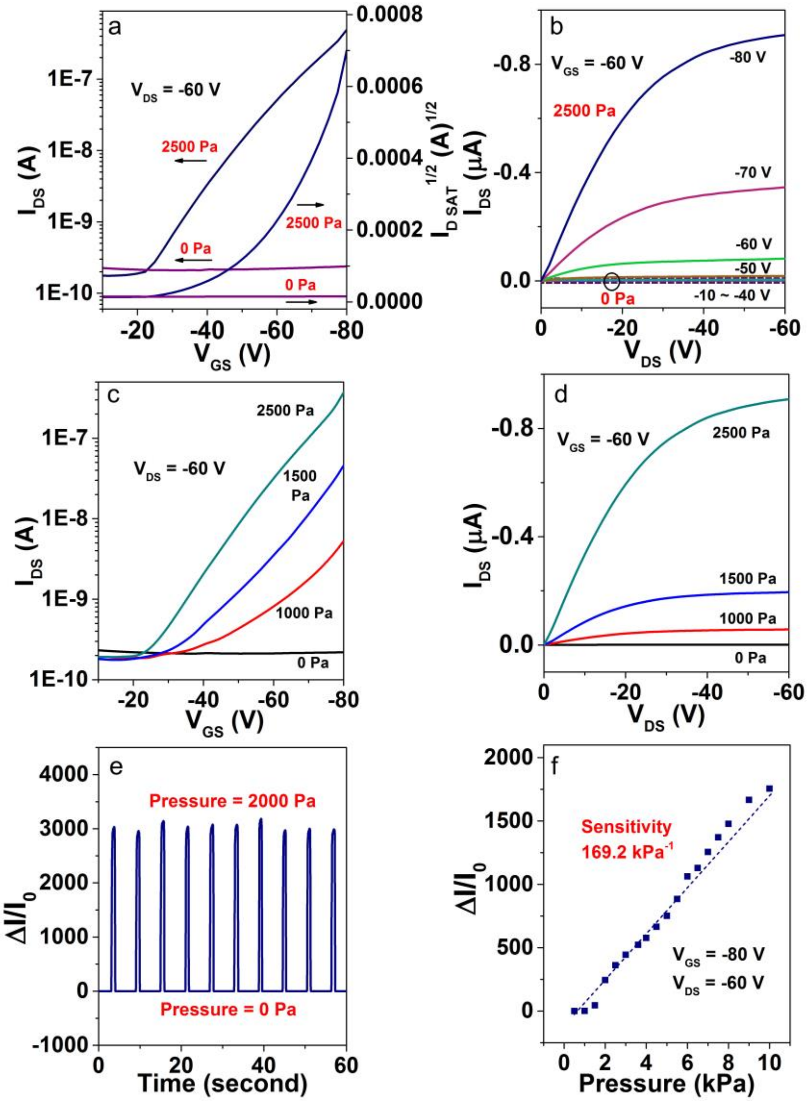

Supplementary Figure7 | Fundamental electric response and performance data for the PDPP3T based SGOTFTs with PMMA dielectric layer. (a) Transfer and (b) output curves of a PMMA dielectric based SGOTFT sensor taken at a pressure of 0 Pa and 2500 Pa. (c) Transfer and (d) output curves of the SGOTFT sensor taken at different applied pressures. (e) Dynamic pressure response of the source-drain current under a pressure of 2000 Pa, at constant voltage V_{DS}=–60 V and V_{GS}=–80 V. (f) Pressure response sensitivity of the source-drain current at constant voltage V_{DS}=–60 V and V_{GS}=–80 V.

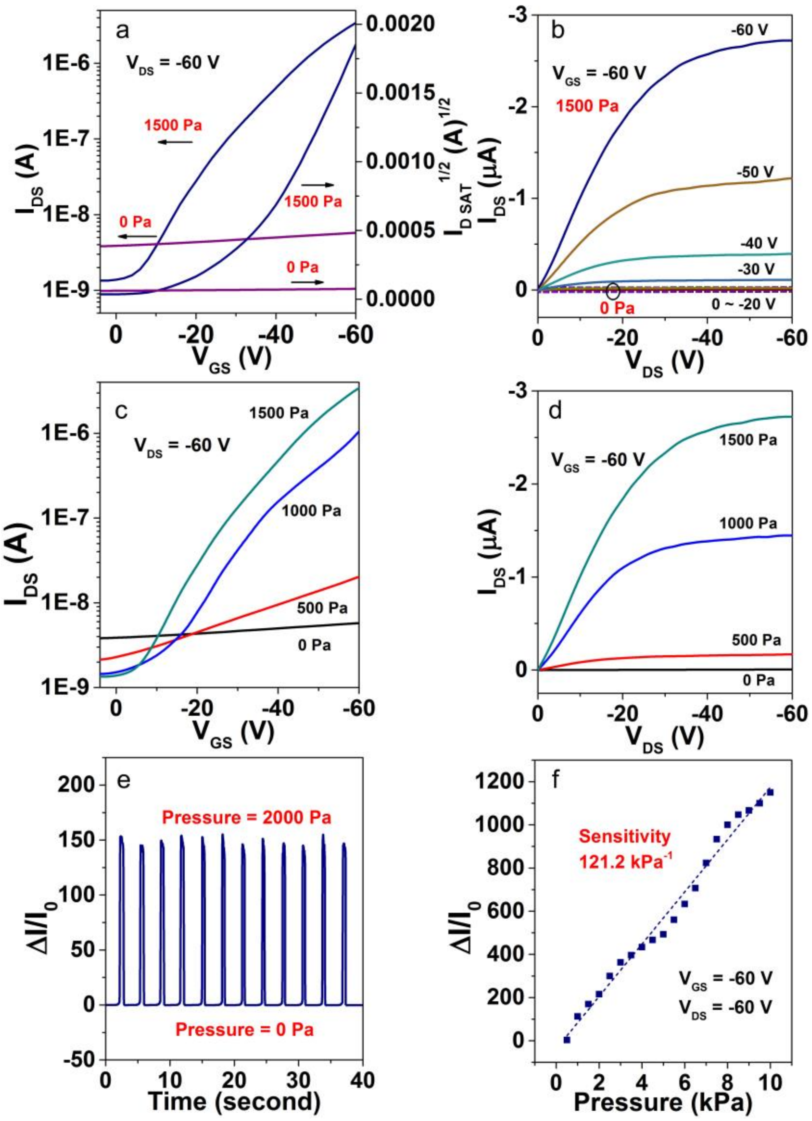

Supplementary Figure 8 | Fundamental electric response and performance data for the PDPP3T based SGOTFTs with PS dielectric layer. (a) Transfer and (b) output curves of a PS dielectric based SGOTFT sensor taken at a pressure of 0 Pa and 1500 Pa. (c) Transfer and (d) output curves of the SGOTFT sensor taken at different applied pressures. (e) Dynamic Pressure response of the source-drain current under a pressure of 2000 Pa, at constant voltage V_{DS}=–60 V and V_{GS}=–60 V. (f) Pressure response sensitivity of the source-drain current at constant voltage V_{DS}=–60 V and V_{GS}=–60 V.

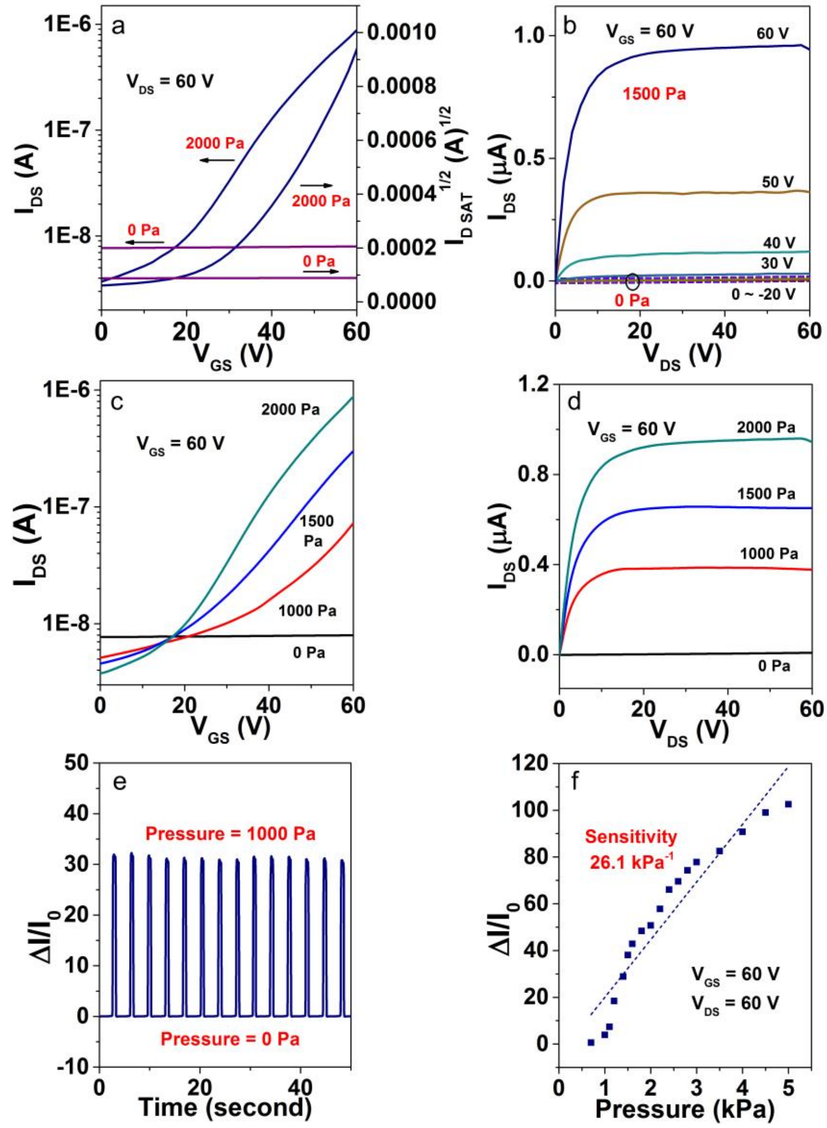

Supplementary Figure 9 | Fundamental electric response and performance data for the NDI3HU-DTYM2 based SGOTFTs with CYTOP dielectric layer. (a) Transfer and (b) output curves of a NDI3HU-DTYM2 based SGOTFT sensor taken at a pressure of 0 Pa and 1500 Pa. (c) Transfer and (d) output curves of the SGOTFT sensor taken at different applied pressures. (e) Dynamic pressure response of the source-drain current under a pressure of 1000 Pa, at constant voltage V_{DS}= 60 V and V_{GS}= 60 V. (f) Pressure response sensitivity of the source-drain current at constant voltage V_{DS}= 60 V and V_{GS}= 60 V.

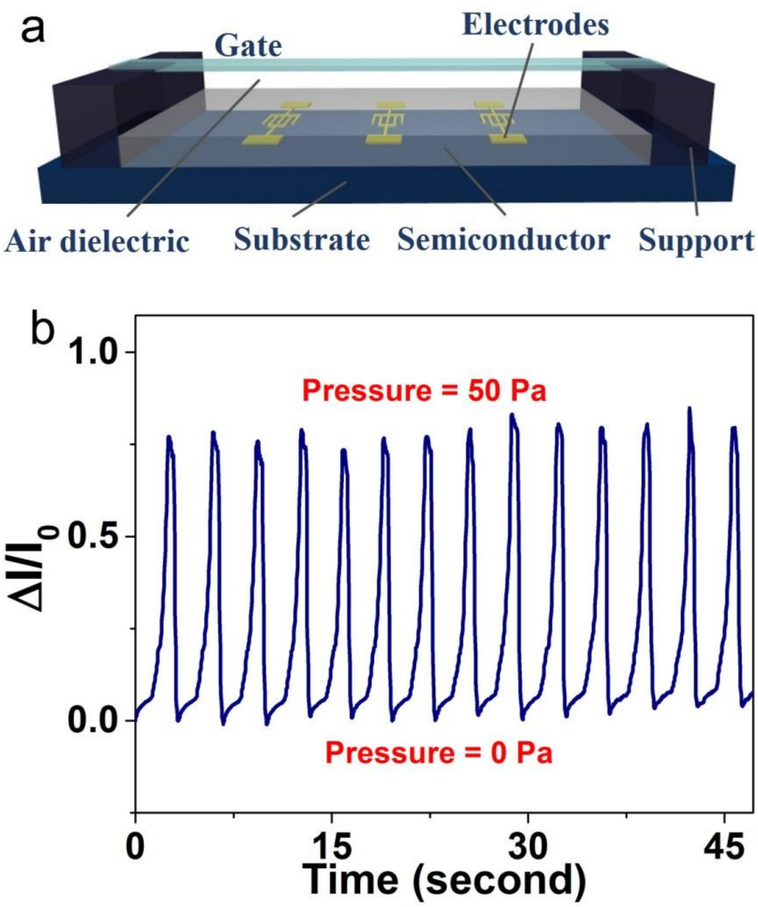

Supplementary Figure 10 | Illustrative schematic and electric properties of SGOTFT without protective dielectric layer. (a) Schematic illustration of the device geometry of a SGOTFT without protective dielectric layer. (b) Dynamic Pressure response of the source-drain current under a pressure of 50 Pa, at constant voltage V_{DS}=–60 V and V_{GS}=–60 V.

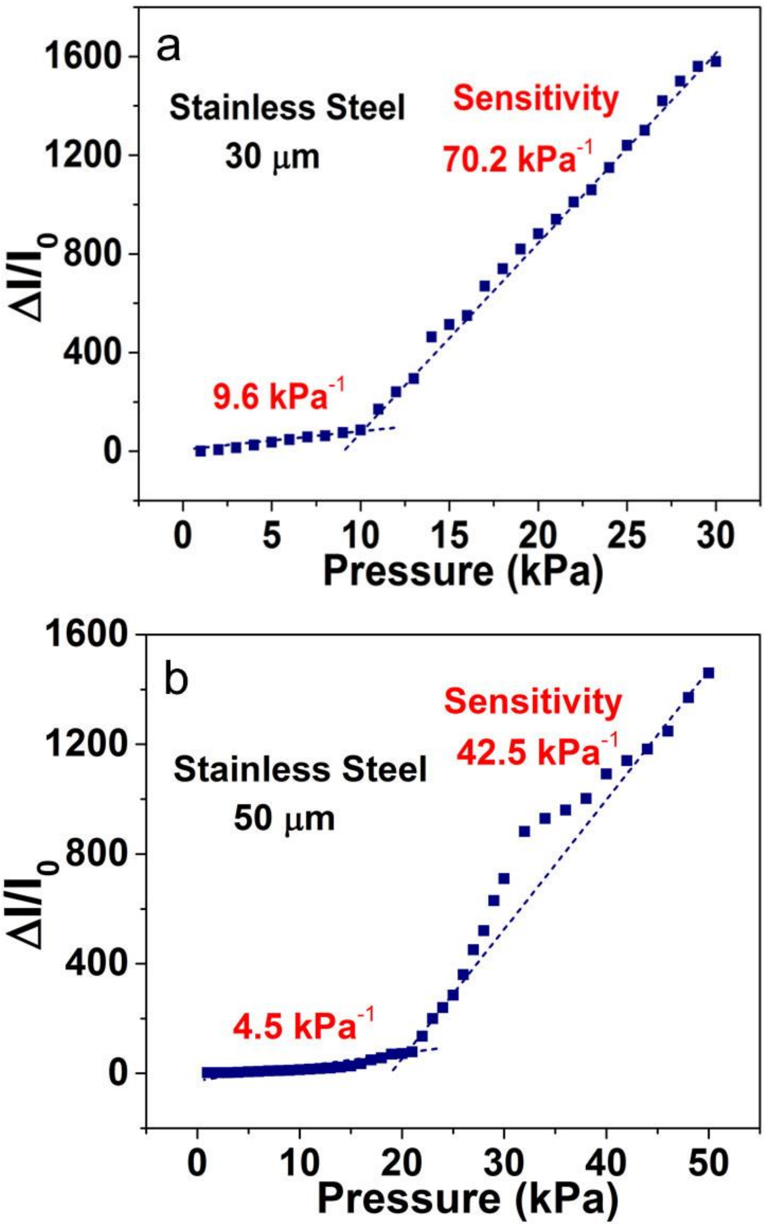

Supplementary Figure 11 | **Electric properties of stainless steel gate based SGOTFTs with different gate Thickness.** Current response of SGOTFTs with a (a) 30 µm and (b) 50 µm thickness stainless steel as the gate electrode, respectively.

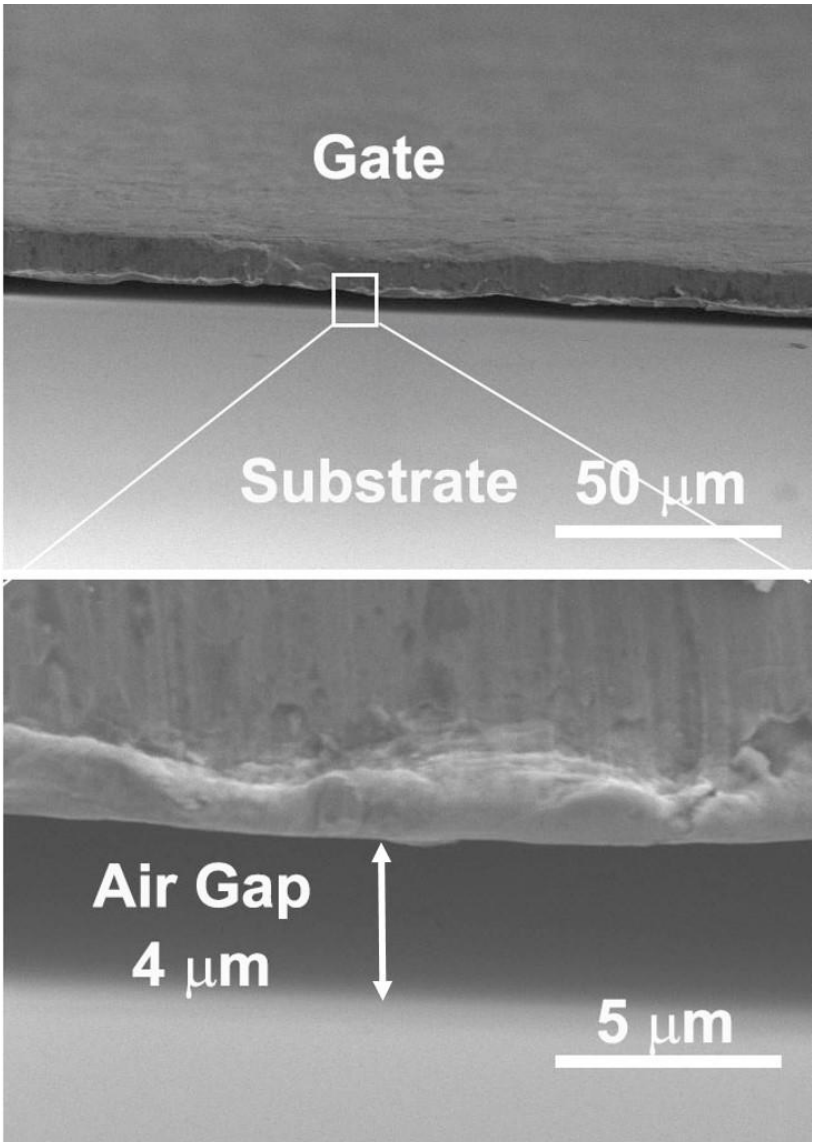

Supplementary Figure 12 | SEM images of a SGOTFT acoustic sensor with an air gap of 4 μm.

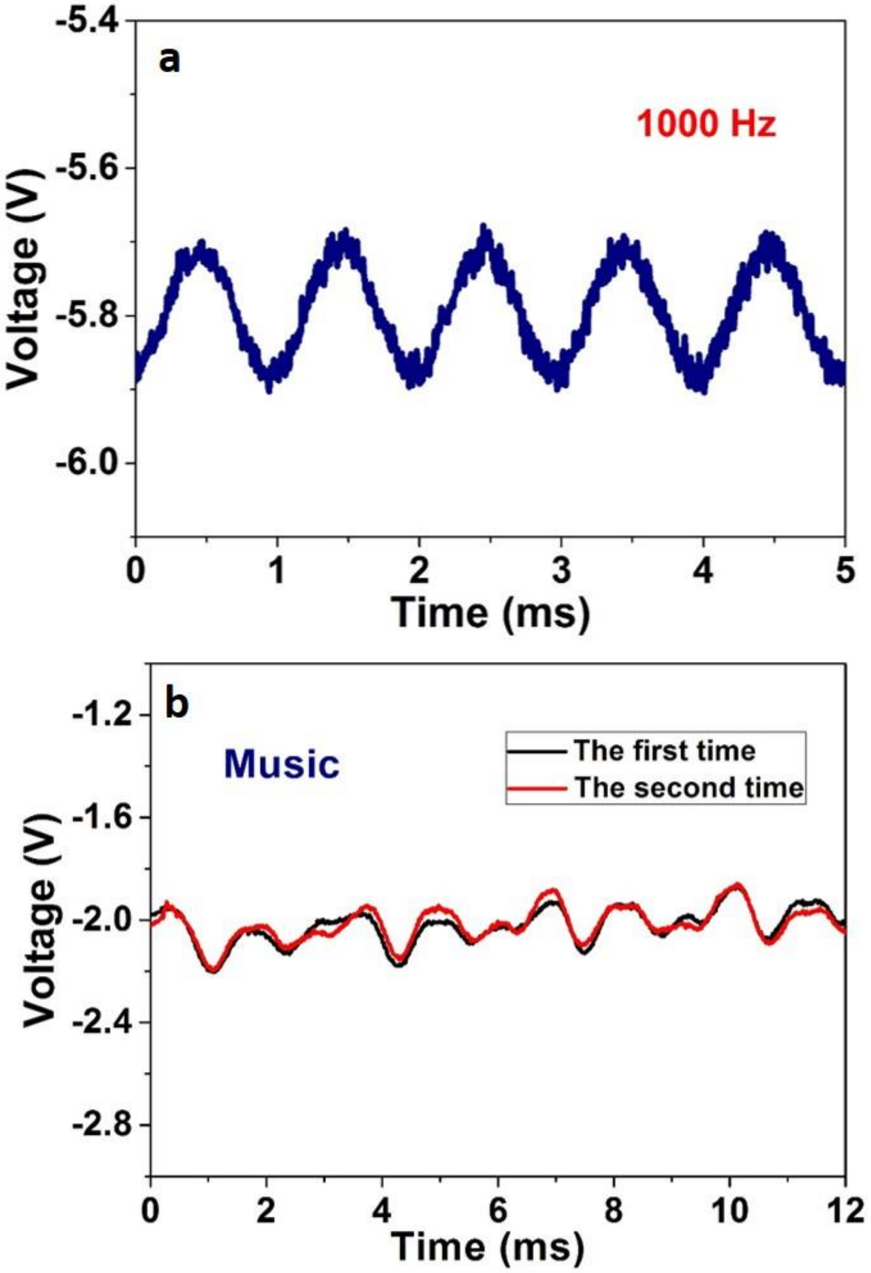

Supplementary Figure 13 | **Acoustic responses.** (a) Response of a sensor, integrated with 2 μm photoresist and 4 μm Al foil suspended gate, to the acoustic wave with a fixed frequency of 1 kHz. (b) Electric signal response of a sensor to the same music for two times.

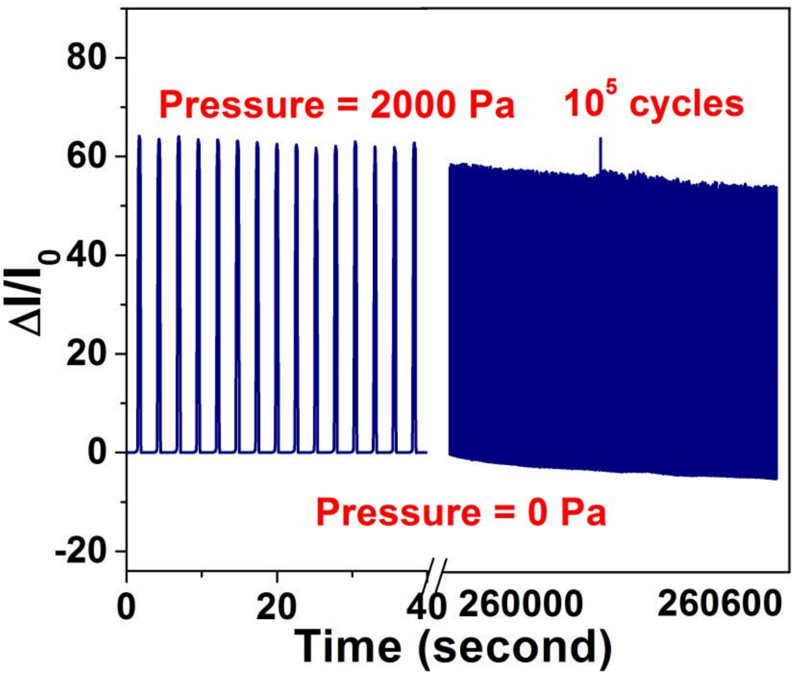

Supplementary Figure 14 | **Stability of NDI3HU-DTYM2 based SGOTFT.** The durability test of a SGOTFT under a pressure of 2000 Pa for 10^5 loading-unloading circles.

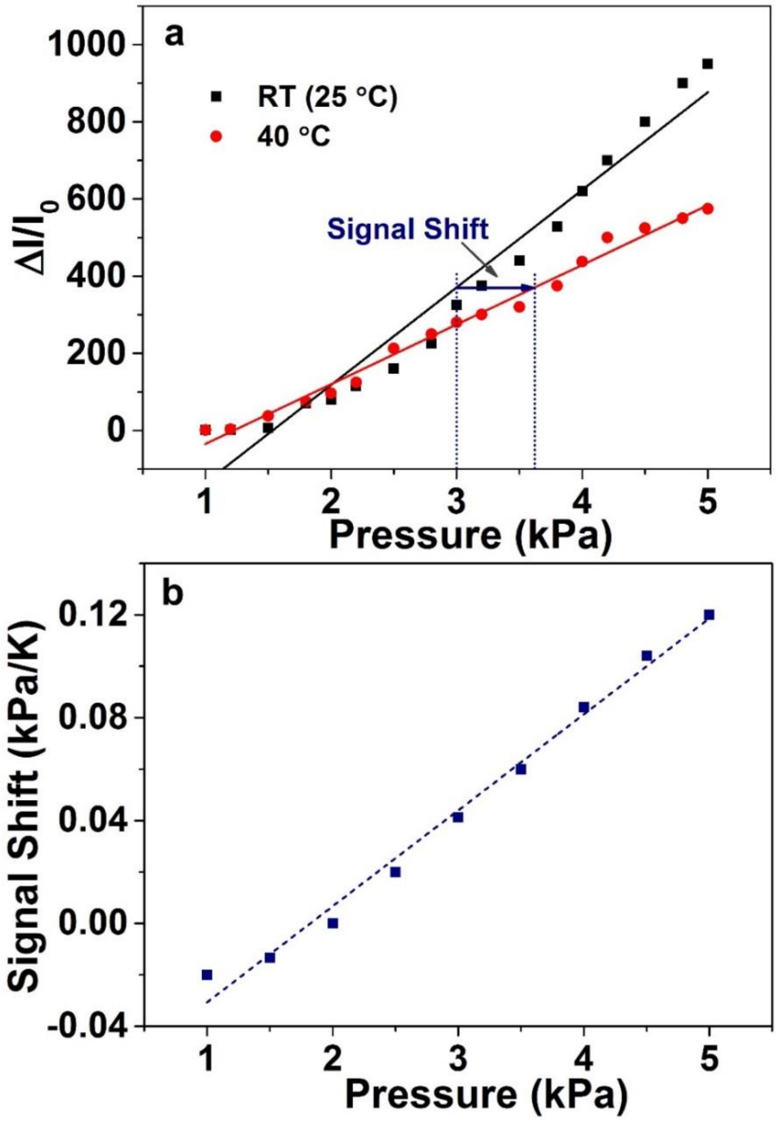

Supplementary Figure 15 | Temperature dependence of pressure sensing responses.

(a) Pressure response sensitivity of the source-drain current at RT and 40 ℃, respectively. (b) Signal shift at a given temperature varies with the applied pressure.

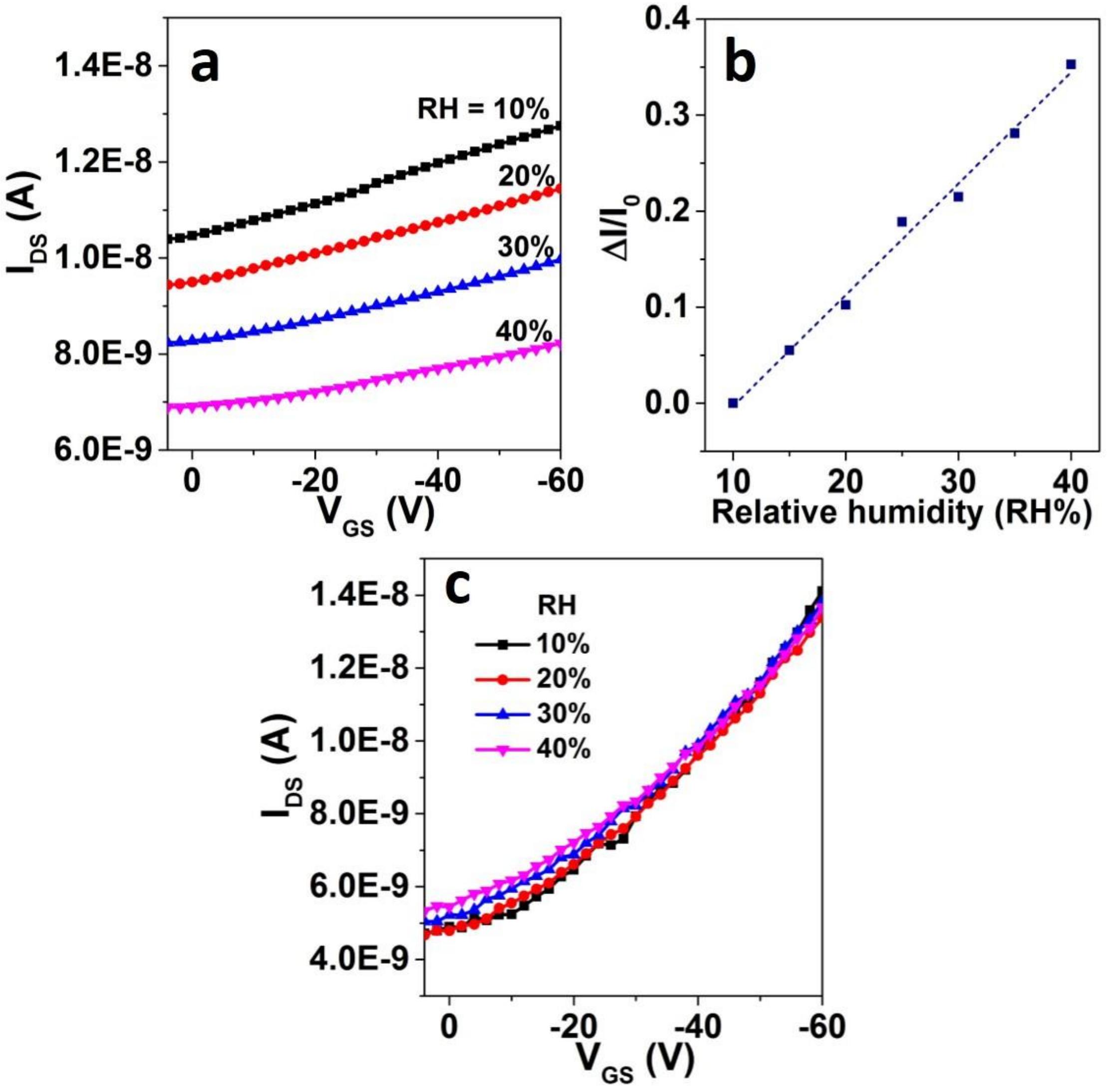

Supplementary Figure 16 | **Relative humidity (RH) responses of the SGOTFTs. (a)** Transfer curves of a SGOTFT sensor taken at a RH of 10%, 20%, 30% and 40%, respectively. (b) RH response sensitivity of the source-drain current at constant voltage V_{DS}=–60 V and V_{GS}=–60 V. (c) Transfer curves of an encapsulated SGOTFT sensor taken at a RH of 10%, 20%, 30% and 40%, respectively.

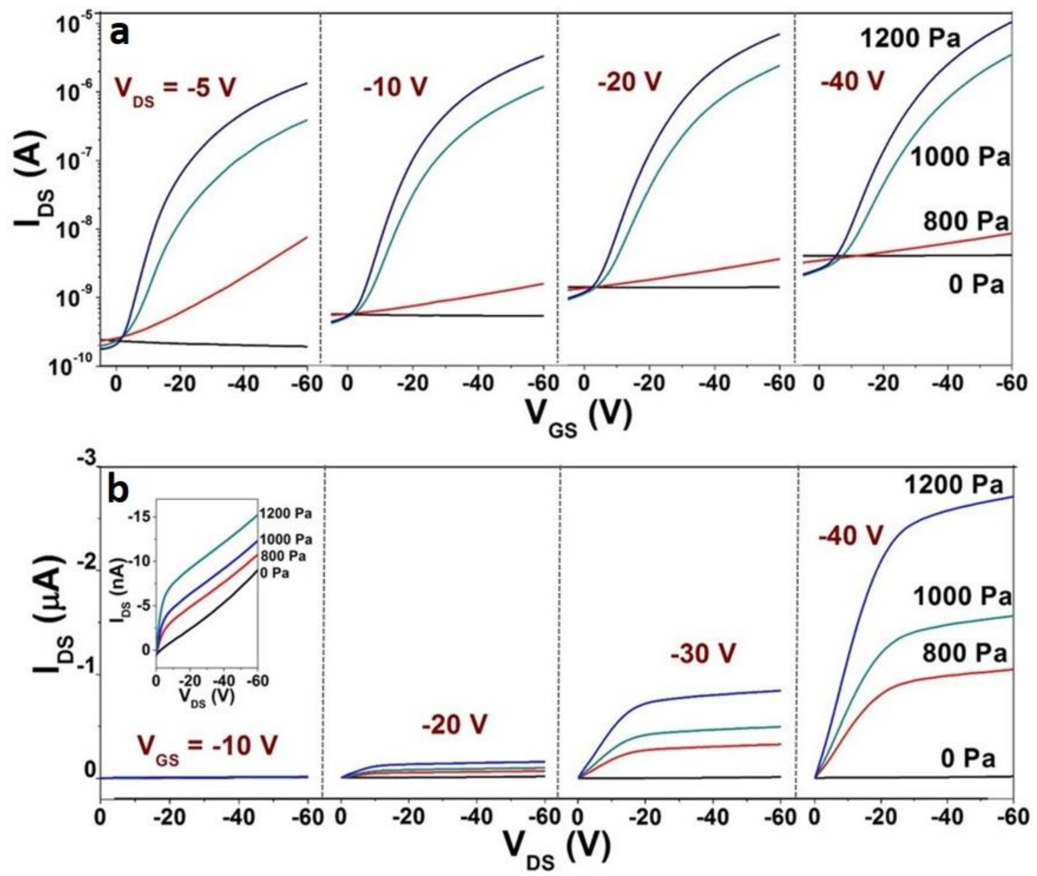

Supplementary Figure 17 | Operating voltage dependent pressure sensing performance. (a) Source-drain voltage dependent transfer curves of a SGOTFT sensor taken at different pressures. (b) Gate voltage dependent output curves of a SGOTFT sensor taken at different pressures. The inset shows the magnified response curves under a V_{GS} of –10 V.

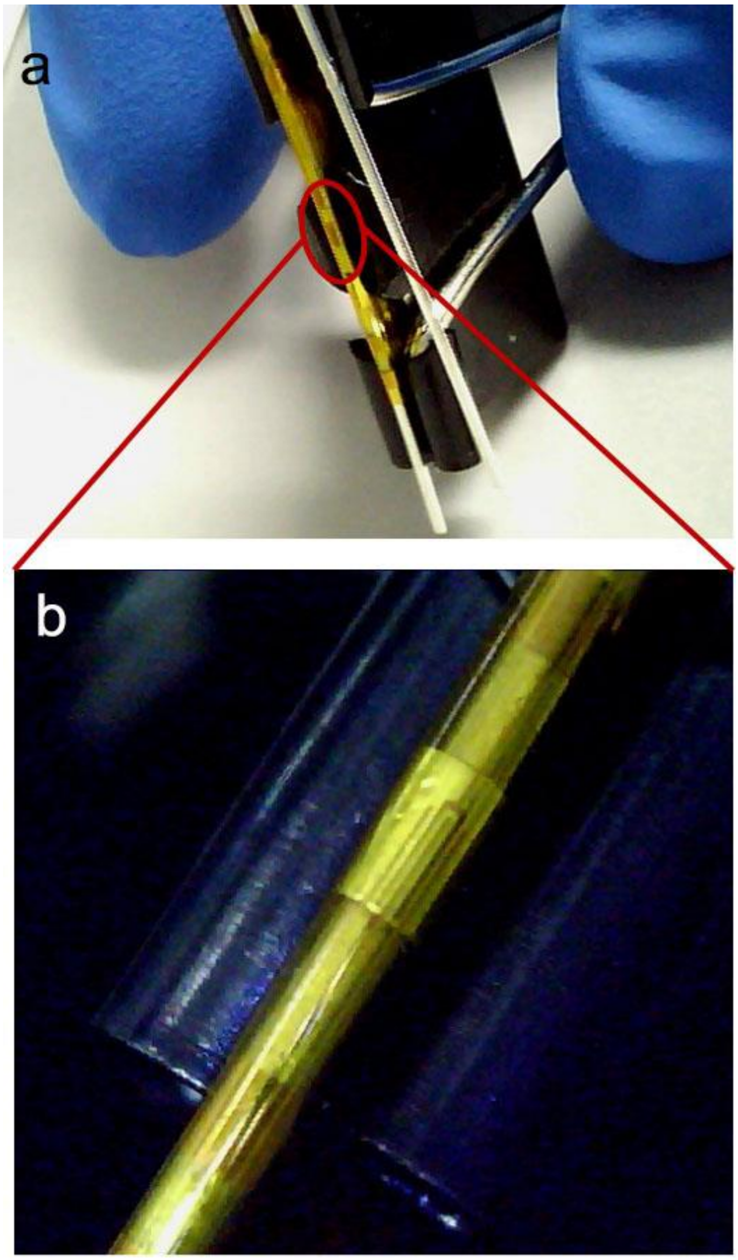

Supplementary Figure 18 | Photographs of the bending test of the SGOTFT sensor. (a) A photograph of SGOTFT devices which are being attached to the terminal of a LED with a bending radii of 0.25 mm. (b) A Close up photograph of the bending devices, a SGOTFT is observed.

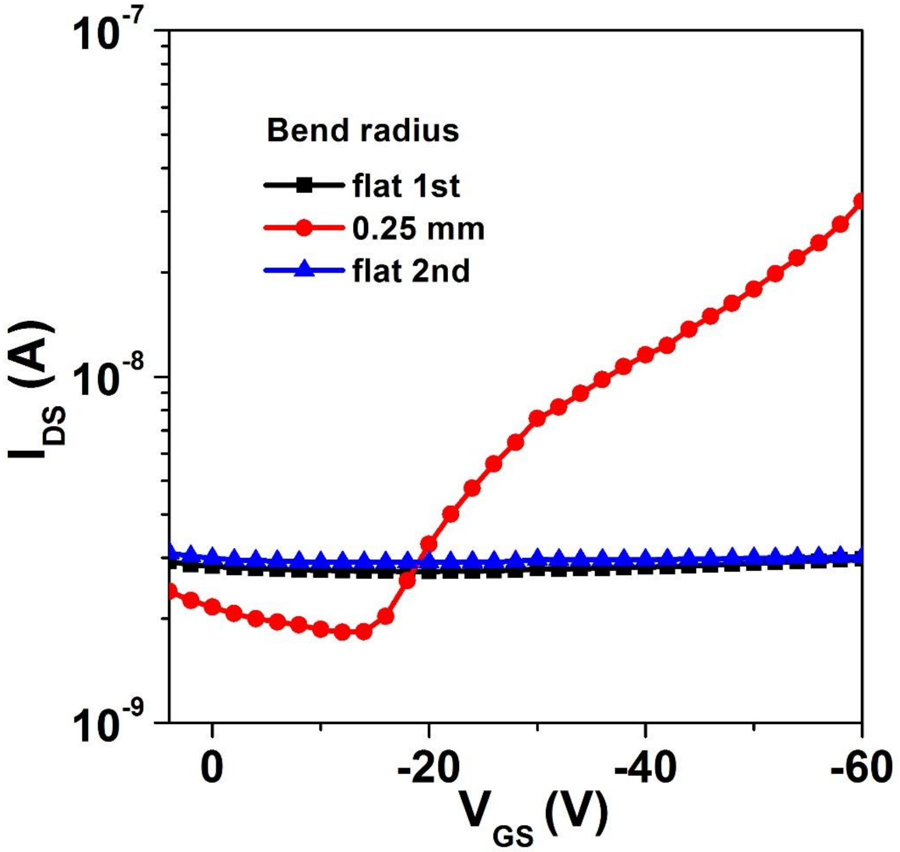

Supplementary Figure 19 | Electric characteristics of the bending test of the SGOTFT sensor. Transfer curves before bending (flat 1st), during bending to 0.25 mm, and after flattening it again (flat 2nd) at constant voltage V_{DS}=–60 V.

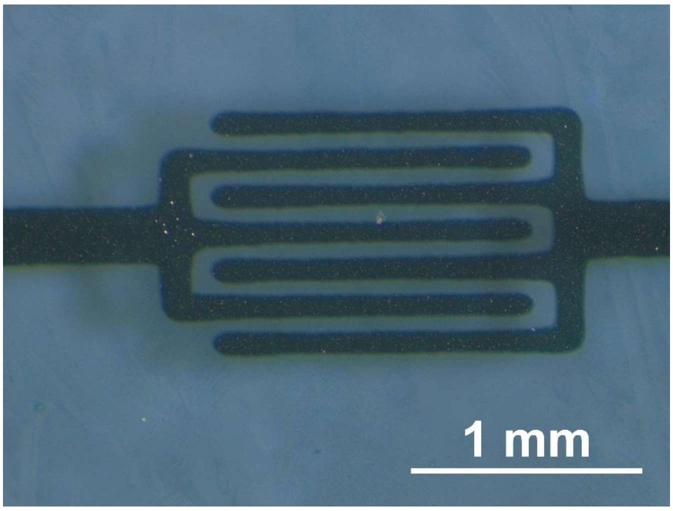

Supplementary Figure 20 | Microscope image of a pixel in the SGOTFT sensor arrays.

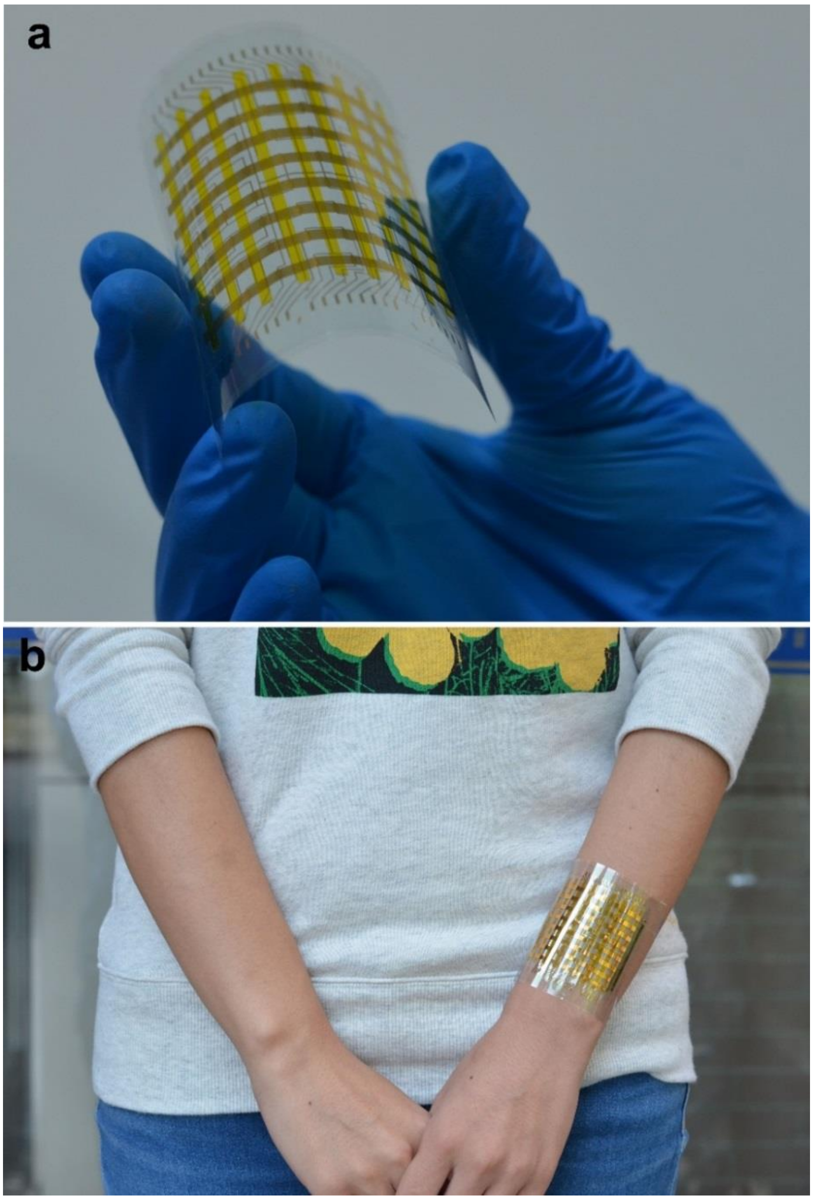

Supplementary Figure 21 | **Photographs of a flexible SGOTFT array.** (a) A photograph of a flexible 8×8 SGOTFT array. (b) A photograph of the array attached to the wrist.

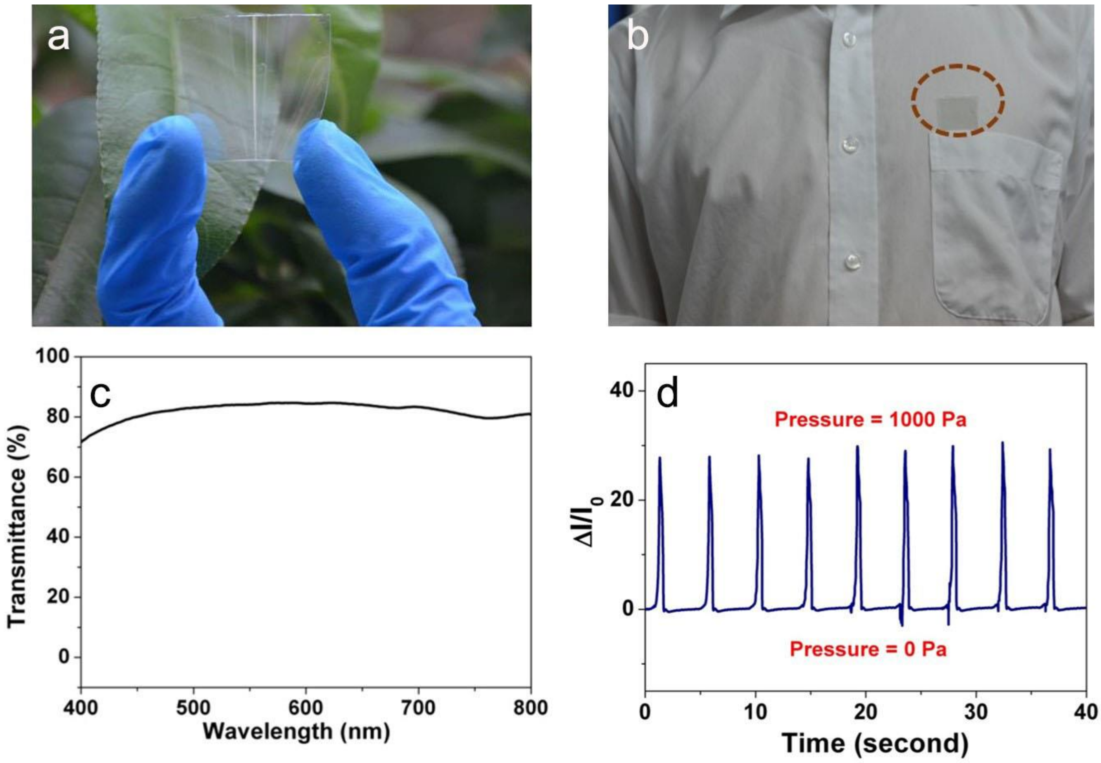

Supplementary Figure 22 | **Transparent SGOTFT pressure sensor.** Photographs of (a) the transparent SGOTFT with ITO serve as the source-drain electrode and gate electrode and (b) the transparent device attached to the clothes. (c) The transmission spectra and (d) dynamic Pressure response of the source-drain current under a pressure of 1000 Pa, at constant voltage V_{DS}=–60 V and V_{GS}=–60 V.

Supplementary Notes

Supplementary Note 1. Mechanism of device operation

The SGOTFT essentially consists of a flexible gate that is suspended over the channel of an OTFT. The structure and equivalent circuit of the SGOTFT are depicted in Fig. 1b and Fig. 1f, respectively. The device can be regarded as a series of two capacitors, the gap capacitor and the protective dielectric capacitor, both of which contribute to the total capacitance (C_{total}) of the SGOTFT per the following equation[1]:

$$C_{total} = \frac{1}{\frac{1}{C_{gap}}+\frac{1}{C_{pd}}} \tag{1}$$

where C_{gap} and C_{pd} represent the gap capacitance and the protective dielectric capacitance, respectively.

The region in the dashed frame (Fig. 1f) is regarded as the classical OTFT structure, where V_{Gint} is defined as the effective input gate voltage to the OTFT. The value of V_{Gint} is affected by the gate voltage V_{GS} and by C_{gap} and C_{pd}, collectively. Their relationship can be expressed as follows:

$$V_{Gint} = \frac{V_{GS}}{1+\frac{C_{pd}}{C_{gap}}} \tag{2}$$

given the equation

$$C = \frac{\varepsilon_r \varepsilon_0 A}{\mathrm{d}} \tag{3}$$

where C represents the capacitance, ε_0 is the absolute dielectric constant, ε_r is the relative dielectric constant of the dielectric material, A is the area of overlap between the gate and the device and d is the thickness of the dielectric layer. According to Eq. (3), C_{pd} is a constant that is determined by the property of the protective dielectric

layer, whereas C_{gap} is a variable capacitance that is dependent on the variable thickness of the air gap (d_{gap}), which depends on the position of the suspended gate, which is, in turn, affected by the external pressure applied to it.

For a classical OTFT, the drain current in the saturation region can be expressed as follows[2]:

$$I_{DS} = \frac{W\mu C_{pd}}{2L}(V_{Gint} - V_T)^2 \qquad (4)$$

where I_{DS} represents the drain current; L and W represent the channel length and width, respectively, of the OTFT device; μ is the mobility of the carriers; and V_T is the threshold voltage. Substitution of equations (2) and (3) into equation (4) yields

$$I_{DS} = \frac{W\mu C_{pd}}{2L}\left(\frac{V_{GS}}{1+\frac{C_{pd}d_{gap}}{\varepsilon_{air}\varepsilon_0 A}} - V_T\right)^2 \qquad (5)$$

where ε_{air} is the relative dielectric constant of air and is generally considered to have a constant value of 1. According to this equation, I_{DS} changes as d_{gap} varies.

For a typical SGOTFT, when an external pressure (P) is applied to the suspended gate, d_{air} and C_{gap} will change as a function of the imposed pressure. According to Eq. (5), a pressure-dependent drain current I_{DS} is produced at a constant V_G input. Therefore, the pressure-signal input is transformed into a current-signal output by the SGOTFT, thereby realizing the detection of external pressure.

In addition to the external mechanical force (F_{extern}) applied to the suspended gate, there an electrostatic force (F_{elec}) between the gate and the OTFT channel that is proportional to C_{gap} and causes the gate to deflect toward the channel. However, an opposing force (F_{opp}) produced by the pulling force (F_p) blocks the motion of the

suspended gate. Thus, the deformation distance (y) is dependent on the effective force (F_{eff}), which contains components of all three forces. F_{eff} is balanced by the restoring force (F_{re}), which drives the restoration of the deformable gate.

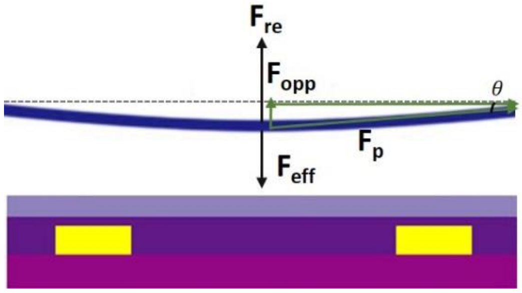

$$F_{eff} = F_{re} = F_{extern} + F_{elec} - F_{opp} = ky \quad (6)$$

$$F_{opp} = F_p sin\theta \quad (7)$$

The effect of the electrostatic force can be neglected in an SGMOSFET with an air gap larger than approximately 60 nm[3]. Because the air gap ranges from 4 to 40 μm when the distance between the support layers is approximately 4 to 10 mm, θ is so small that any changes in θ induced by the variation of d_{gap} can be neglected. Thus, $F_p sin\theta$ can be considered to be a constant (b) upon the application of a pressure bias. Therefore, Eq. (6) can be simplified to

$$F_{extern} = ky + b \quad (8)$$

where k is the rigidity of the suspended gate, which can be calculated from the gate geometry and dimensions as follows:

$$k = \frac{192EI}{l_G^3} \quad (9)$$

$$I = \frac{w_G h_G^3}{12} + \frac{h_G w_G^3}{12} \quad (10)$$

where I is the bending moment of inertia of the rectangular gate, E is the Young's

modulus and h_G, l_G and w_G are the gate thickness, length and width, respectively. According to Eq. (9) and Eq. (10), under a constant external force, the deformation distance (y) that determines the change in the signal of the SGOTFT pressure sensor is inversely proportional to the rigidity of the gate. Decreasing the rigidity of the suspended gate would improve the sensitivity of the pressure-sensing device. Thus, elastic materials of low modulus are preferred for use as suspended gates. Controlling the dimensions of the suspended gate is another effective strategy for improving the sensitivity of the pressure sensor; long, narrow, ultra-thin films tend to be ideal candidates for the construction of ultra-sensitive SGOTFT pressure sensors. It can therefore be concluded that SGOTFTs can serve as a platform for the fabrication of tunable pressure sensors with optimized sensitivities within a desired pressure range, thereby fulfilling the requirements of various practical applications in different areas.

Supplementary Note 2. Calculation of sound pressure

A multi-functional sound level meter (AWA6228-6) was used to measure the sound level produced by a speaker in units of decibels (dB). Using these values, the sound pressure (P_1) was calculated following the equation expressed below:

$$P_1 = P_0 10^{\frac{L_{dB}}{20}} (Pa) \quad (11)$$

where P_0 is a reference sound pressure of 20 μPa and L_{dB} is the measured sound pressure level.

Supplementary References

1 Dai, C. L., Tai, Y. W. & Kao, P. H. Modeling and Fabrication of Micro FET Pressure Sensor with Circuits. *Sensors (Basel)* **7**, 3386-3398 (2007).

2 Dai, C. L., Lu, P. W., Wu, C. C. & Chang, C. Fabrication of Wireless Micro Pressure Sensor Using the CMOS Process. *Sensors (Basel)* **9**, 8748-8760 (2009).

3 Sallese, J., Grabinski, W., Meyer, V., Bassin, C. & Fazan, P. Electrical modeling of a pressure sensor MOSFET. *Sens. Actuators.* **94**, 53-58 (2001).
